# Ecology and genomics of an important crop wild relative as a prelude to agricultural innovation

**DOI:** 10.1038/s41467-018-02867-z

**Published:** 2018-02-13

**Authors:** Eric J.B. von Wettberg, Peter L. Chang, Fatma Başdemir, Noelia Carrasquila-Garcia, Lijalem Balcha Korbu, Susan M. Moenga, Gashaw Bedada, Alex Greenlon, Ken S. Moriuchi, Vasantika Singh, Matilde A. Cordeiro, Nina V. Noujdina, Kassaye Negash Dinegde, Syed Gul Abbas Shah Sani, Tsegaye Getahun, Lisa Vance, Emily Bergmann, Donna Lindsay, Bullo Erena Mamo, Emily J. Warschefsky, Emmanuel Dacosta-Calheiros, Edward Marques, Mustafa Abdullah Yilmaz, Ahmet Cakmak, Janna Rose, Andrew Migneault, Christopher P. Krieg, Sevgi Saylak, Hamdi Temel, Maren L. Friesen, Eleanor Siler, Zhaslan Akhmetov, Huseyin Ozcelik, Jana Kholova, Canan Can, Pooran Gaur, Mehmet Yildirim, Hari Sharma, Vincent Vadez, Kassahun Tesfaye, Asnake Fikre Woldemedhin, Bunyamin Tar’an, Abdulkadir Aydogan, Bekir Bukun, R. Varma Penmetsa, Jens Berger, Abdullah Kahraman, Sergey V. Nuzhdin, Douglas R. Cook

**Affiliations:** 1 Department of Biological Sciences and International Center for Tropical Botany, Florida International University, Miami, FL 33199 USA; 2 Department of Plant and Soil Science, University of Vermont, Burlington, VT 05405 USA; 3 Department of Plant Pathology, University of California Davis, Davis, CA 95616 USA; 4 Department of Biological Sciences, University of Southern California, Los Angeles, CA 90089 USA; 5 Faculty of Agriculture, Dicle University, Diyarbakir, 21280 Turkey; 6 Institute of Biotechnology, Addis Ababa University, Addis Ababa, 32853 Ethiopia; 7 Ethiopian Institute of Agricultural Research, Addis Ababa, P.O. Box 2003 Ethiopia; 8 Hawassa University, Hawassa, 005 Ethiopia; 9 Oromia Agricultural Research Institute (OARI), Addis Ababa, P.O. Box 81265 Ethiopia; 10 Department of Applied Mathematics, Peter the Great St. Petersburg Polytechnic University, St. Petersburg, 195251 Russia; 11 Department of Plant Sciences, Quaid-i-Azam University Islamabad, Islamabad, 45320 Pakistan; 12 Department of Plant Sciences, University of Saskatchewan, Saskatoon, SK S7N5A8 Canada; 13 Faculty of Pharmacy, College of Agriculture, Dicle University, Diyarbakır, 21280 Turkey; 14 Department of Field Crops, Faculty of Agriculture, Harran University, Sanliurfa, 63100 Turkey; 15 Department of Botany, University of Florida, Gainesville, FL 33181 USA; 16 Department of Plant Biology, Michigan State University, East Lansing, MI 48823 USA; 17 Black Sea Agricultural Research Institute, Samsun, P.O. Box 39 Turkey; 18 International Crops Research Institute for the Semi-Arid Tropics, Patancheru, 502324 Telangana India; 19 Department of Biology, Gaziantep University, Gaziantep, 27310 Turkey; 20 Central Research Institute for Field Crops (CRIFC), Ankara, 06042 Turkey; 21 Commonwealth Scientific and Industrial Research Organization (CSIRO), Agriculture and Food, Perth, 6014 WA Australia

## Abstract

Domesticated species are impacted in unintended ways during domestication and breeding. Changes in the nature and intensity of selection impart genetic drift, reduce diversity, and increase the frequency of deleterious alleles. Such outcomes constrain our ability to expand the cultivation of crops into environments that differ from those under which domestication occurred. We address this need in chickpea, an important pulse legume, by harnessing the diversity of wild crop relatives. We document an extreme domestication-related genetic bottleneck and decipher the genetic history of wild populations. We provide evidence of ancestral adaptations for seed coat color crypsis, estimate the impact of environment on genetic structure and trait values, and demonstrate variation between wild and cultivated accessions for agronomic properties. A resource of genotyped, association mapping progeny functionally links the wild and cultivated gene pools and is an essential resource chickpea for improvement, while our methods inform collection of other wild crop progenitor species.

## Abstract (web summary)

Domestication reduces genetic diversity and constrains crop improvement. Here the authors identify factors that shaped species diversity in the wild progenitors of chickpea, and produce wild introgression populations that increase diversity for breeding by ~100-fold, including traits of agronomic relevance.

## Introduction

Wild relatives are the primary reserve of a crop’s genetic variation^1,2^. Despite their potential value in meeting the challenges of modern agriculture, few systematic, range-wide collections of wild relatives exist for any crop species, and even the available wild genetic resources are widely under-utilized for crop improvement^1,3–5^. Only recently have advances in genomics and phenotyping made it realistic to identify and introgress wild traits at scales necessary to impact agriculture broadly, including complex traits that underpin crop yields^3,5,6^.

In natural populations, micro-heterogeneity of habitats can maintain variation at small scales, while variation among environmentally diverse but locally homogenous sites can drive population differentiation and local adaptation^7^. Thus, collections that span a range of physical scales and cover the species’ geographic range are most likely to capture the greatest breadth of useful adaptations. Importantly, the environments into which domestication occurred approximately 10,000 years ago are very different from those confronting modern agriculture, making it likely that certain wild adaptations useful in today’s agriculture were not selected during historical domestication. Moreover, because populations of many wild relatives are under threat from habitat loss and degradation, there is an urgent need to collect and conserve crop wild relatives (CWRs) and to do so using methods that maximize genetic and environmental breadth. Such collections need to be characterized in ways that facilitate their use to solve current and future agricultural challenges^1^.

Mesopotamia, within the so-called ‘‘Fertile Crescent’’, is the source of several prominent crop species, including wheat, barley, flax, lentil, peas, fava bean, and chickpea^8^. Chickpea (*Cicer arietinum* L.) is a legume of considerable importance to food security, especially in India, Pakistan and Ethiopia, where ~22% of the world’s population depends on chickpea and other legumes as a primary source of nutritional nitrogen. Chickpea and its immediate wild relatives are predominantly selfing diploids with eight chromosomes and ~750 MB genomes^9^. *C. reticulatum* is the immediate wild progenitor of chickpea, restricted to a few provinces of southeastern Anatolia in modern day Turkey. Despite this narrow geographical range, the region has a variety of soil substrates and a significant elevational gradient spanning over 1000 m, which bring differences in climatic conditions that could drive distinct local adaptations.

In chickpea, a crop with limited genetic variation^9–11^ and very minimal collections of its immediate wild relatives (e.g., *C. reticulatum* Ladiz and its sister species *C. echinospermum* P.H. Davis^12^), new collections may enable breeders to address pressing needs, such as increased resilience to drought, heat and cold, increased seed nutrient density, reduced dependence on inputs, and resistance to biotic stress. Towards these ends, we combine ecological principles to guide collection across the full range of habitats where wild chickpea occurs, using population genomics and phenotyping to characterize the collection, and genetic crosses to generate pre-breeding populations. The collection we report contains greatly expanded genomic diversity and a range of traits of potential agronomic importance. In doing so, we implement a rational approach that can guide the construction and utilization of CWR collections in other crops.

## Results

### The rationale for and scope of the collection

**Fig. 1:**
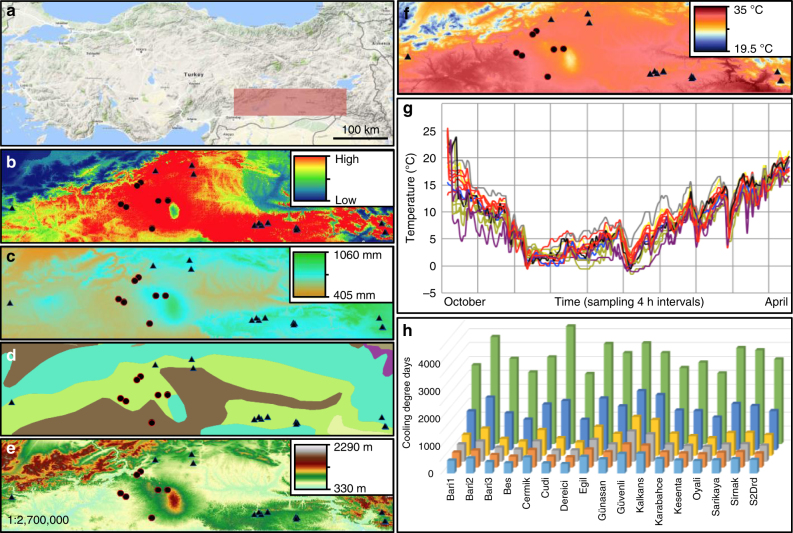
Environmental variation across the native range of the wild relatives of chickpea. **a** Topographic map of Turkey with the collection region featured in **b**–**f** highlighted in red. Map data obtained from @2018 Google, ORION-ME. **b** Bioclimatic model of the likelihood of species distribution, combined for both *C. reticulatum*—black rectangle and *C. echinospermum*—black circles. **c** Total annual precipitation across southeastern Anatolia. **d** Major soil types: Calcisols—brown circles; Kastanozems—purple circles; Leptosols—blue-green circles; Luvisols—lime-green circles; Vertisols—yellow circles. **e** Elevation across southeastern Anatolia. **f** Summer maximum temperature in southeastern Anatolia. Panels **b**–**f** were generated using Maxent open-source software^61^. **g** Variation across collection sites in maximum temperature, recorded at 4 h intervals 5 cm below the soil surface with ibutton data loggers. Data are shown for 22 October 2013 through 21 April 2014, which encompasses winter and through late vegetative growth of the wild species. **h** Modified photo-thermal units (i.e., cooling degree days) at each site integrated over the growing season from 1 November 2013 until 15 May 2014. November—light blue squares; December—orange squares; January—gray squares; February—yellow squares; March—blue squares; April–May—green squares

A bioclimatic model in DIVA-GIS identified likely locations of wild species in a ~60,000 km^2^ area that we explored over 53 days in 2013 (Fig. 1). With the aid of local shepherds, who readily recognized the two species, our survey yielded 21 field sites, 371 accessions as seed and 839 plants as DNA, and as described below, resulted in a large increase in available genomic and phenotypic diversity (Supplementary Data 1). Single seed descent was used to establish an immortalized resource of wild germplasm, now in the international genebank system^13^, representing a ninefold and 21-fold increase in available, non-redundant *C. echinospermum* and *C. reticulatum* accessions. Two *C. bijugum* Rech. f. field sites were included in the analysis because they were within the spatial range of the other species, and because *C. bijugum* serves as a useful evolutionary outgroup that is related to but genetically incompatible with cultivated chickpea.

### Site environments

**Fig. 2:**
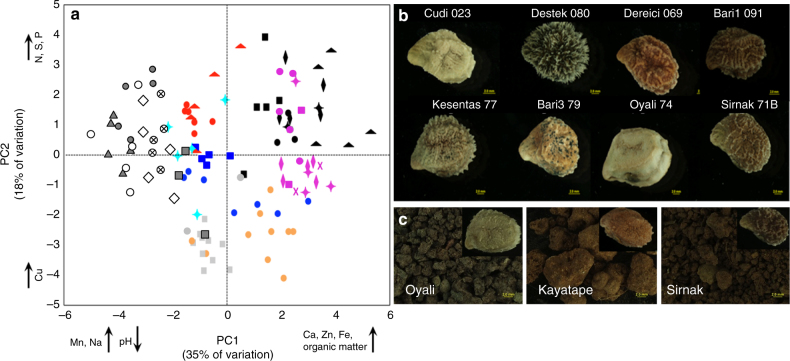
Variation in soil chemistry and cryptic matching of soils by wild *Cicer* seed color. **a** Principal component analysis of variation in 18 soil factors, with five samples from each collection site. Loading on the first two axes explains 35 and 18% of the variation. Factors with high loading (above 1.0) are indicated on the axes. Shapes and colors correspond to distinct field sites. *C. echinospermum* sites: Cermik—open circle with “X”; Günaşan—gray triangle; Güvenli—open diamond; Karabahce—open circle; S2Drd—gray circle; Ortanca—gray square. Common colors among *C. reticulatum* field sites reflect adjacency of locations: Baristepe1—pink circle; Baristepe1 agricultural soil—pink “X”; Baristepe2—pink star; Baristepe3—pink diamond; Baristepe3 agricultural soil—pink square; Beşevler—black triangle; Dereiçi—black diamond; Sarikaya—black square; Savur—black circle; Egil—red triangle; Kalkan—red circle; Kayatape—black star; Kesentaş—light blue star; Oyali—orange circle; Cudi A/B—royal blue circle; Şırnak—royal blue square. *C. bijugum*: Dargrecit—gray circle; Kilavuz—gray square. **b** Variation in seed color and texture. Seeds of eight distinct lines representing the range of color and texture variation are shown. **c** Color matching of seeds and dry soils. Seeds and soils from Oyalı, Kayatape, and Şirnak are shown

Aspects of soil chemistry, temperature, relative humidity, and climate were recorded at multiple spatial scales, and analyzed both within and among sites (Fig. 1 and Fig. 2). The WorldClim database^14^ provided a coarse view of temperature and rainfall patterns among sites (resolution ~1 km^2^) and included prior years. To evaluate variation within sites, soil samples were collected from up to ten plant microsites at each field location and analyzed for 18 aspects of soil chemistry and texture (Fig. 2a and Supplementary Data 2). A similar spatial scheme was used to assess year-long variation in temperature by placing thermochron and hygrochron data loggers just under the soil surface (upper 5 cm) and sampling the environment at 4 h intervals (Fig. 1f). Temperature profiles were converted into modified photo-thermal units, which varied significantly among sites (Fig. 1g, Supplementary Table 1) and are akin to cumulative degree-days that drive plant development and phenological traits^15^.

**Fig. 3:**
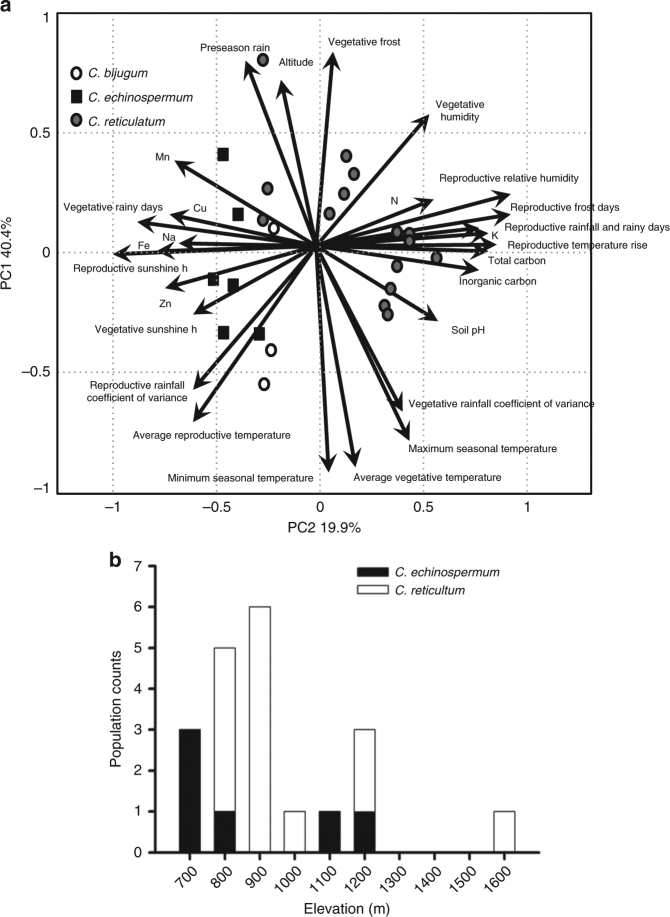
Elevation is an important axis of environmental variation for wild *Cicer*. **a** Principal components analysis of 18 soil factors and 19 bioclimatic variables for each collection site. Arrows indicate loadings of each variable, while points indicate collection sites. **b** Elevation of collection sites

This collection spans a large elevational gradient and associated climatic and soil-type clines. *C. echinospermum* populations generally occur at lower elevations than *C. reticulatum* (740–1264 m versus 915–1695 m, Fig. 3b), with the former on relatively homogeneous geologically derived basaltic soils and the latter on biologically derived limestone and sandstone soils that vary among sites (Supplementary Fig. 1). Temperatures are higher and rainfall is lower on average in *C. echinospermum* sites (538–669 mm average yearly rainfall) compared to *C. reticulatum* sites (545–848 mm), but we also observed significant variation among sites within species. The three most easterly and highest elevation *C. reticulatum* sites are distinguished by having the lowest monthly average temperatures (−4.8 to −2.2 °C versus −2.2 to −0.6 °C) in winter, more frequent vegetative phase frost in spring, and the highest annual rainfall (804–848 mm versus 538–735 mm for the other *C. reticulatum* sites).

A combined principal component analysis (PCA) of climate, elevation, and soil chemistry explained 75% of variation across all variables in the first three axes (Fig. 3). PC1 (40.4% variance) discriminated among *Cicer* species, with primary influences from rainfall, temperature and sunshine, soil micronutrients and fertility (N, K, C) and pH, indicative of broadly different ecological niches for the wild species. In particular, *C. reticulatum* field sites were separated from *C. echinospermum* and *C. bijugum* field sites by less frequent rainfall in the vegetative phase, and a reproductive phase with lower radiation, higher frequency of frost, higher relative humidity and more rapid temperature increase in the summer months. Similarly, *C. reticulatum* soils were more fertile and more alkaline than those where *C. echinospermum* and *C. bijugum* occurred (Fig. 2a). PC2 (19.9% variance), dominated by altitude and its effects on temperature, rainfall distribution and variability, tended to discriminate collection sites within, rather than between species. Higher elevation locations had higher pre-season rainfall, and more variable rainfall throughout both vegetative and reproductive phases. Vegetative phase frost incidence was more frequent at high altitude, while minimum, mean, and maximum temperatures were lower. PC3 (14.7% variance), dominated by contrasting loadings on latitude and longitude, and lesser contributions of altitude and vegetative rainfall, resolved *C. echinospermum* from *C. bijugum*, reflecting their relatively discrete habitat ranges that are potential drivers of species and population divergence.

### Patterns of genetic variation

Genotyping-by-sequencing (GBS) surveyed variation among 1064 individuals, including 985 newly collected wild samples, 56 accessions of *C. arietinum* that encompass the genetic and geographic breadth of the cultigen^16^ and 23 wild reference accessions. Among 879,024 100 bp sequence tags were 148,136 segregating sites. *Cicer arietinum* had the lowest number of polymorphic loci at 25,036, despite having the most number of loci called, highlighting lower genetic diversity in the crop’s germplasm. *C. reticulatum* had significantly greater numbers of polymorphic loci than *C. echinospermum* (136,638 and 88,976 loci, respectively, Supplementary Table 2 and see below for more detailed analyses), reflecting both less diverse local populations and a smaller number of total individuals sampled for *C. echinospermum* and consistent with the narrower range of environments in which the species occurred.

**Fig. 4:**
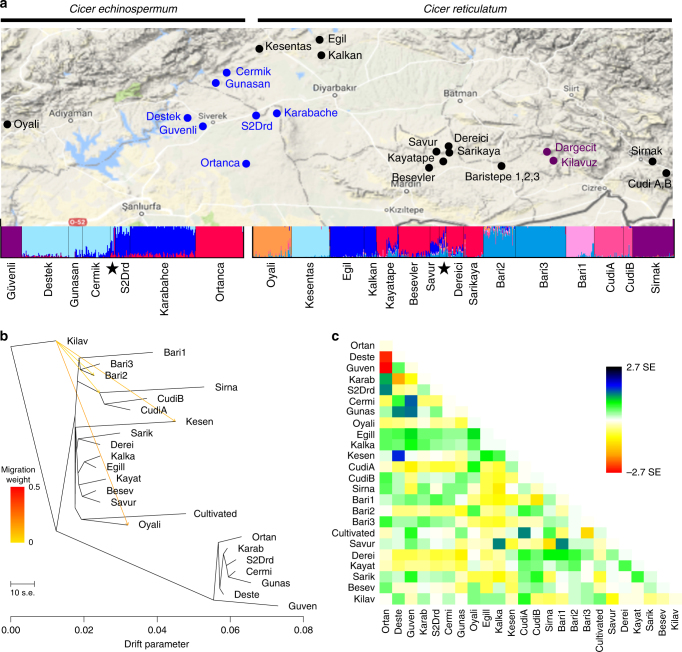
Clustering and patterns of differentiation among wild *Cicer* populations. **a** STRUCTURE assignments (*K* = 13) based on 1064 samples and 16,845 loci SNPs, overlaid on a map of southeastern Anatolia. Collection sites: Guv = Guvenli, Des = Destek, Gun = Gunasan, Cer = Cermik, Kar = Karabahce, Ort = Ortanca, Oy = Oyali, Kes = Kesentas, Kal = Kalkan, Kay = Kayatape, Bes = Besevler, Sav = Savur, Der = Dereici, Sar = Sarikaya, Bar = Baristepe (1,2,3). Wild accessions of *C. echinospermum* and *C. reticulatum* from the USDA National Plant Germplasm System are indicated with a black star, with corresponding accession metadata given in Supplementary Data 1. Map data obtained from @2018 Google, ORION-ME. **b** Best-fitted maximum-likelihood tree displaying relationships between populations with no migration events. Migration edges are colored according to the proportion of source population ancestry found in the sink population. **c** Plot of residuals for the tree depicted in **b**. The color palette depicts the residual covariance between each pair of populations divided by the average standard error across all pairs

Allele frequency data readily resolved the samples into the four species (Fig. 4) using STRUCTURE^17^, nominating eight well-supported populations^18^ among *C. reticulatum* and four among *C. echinospermum*; cultivated nests as one group with *C. reticulatum* and separate from *C. echinospermum* at *K* = 2, but splits into a cultivated-specific group at *K* = 3 (see also Supplementary Fig. 2). Twenty-seven percent of variation segregates within individuals, 27% among individuals within sites, and 46% among sites. Fst varied from as low as 0.03 between *C. echinospermum* populations to as high as 0.77 when comparing between *C. reticulatum* populations, while within *C. reticulatum*, five populations (Kayatepe Savur, Beşevler, Sarıkaya, and Dereiçi) exhibit the highest pairwise distances and comprise an extended, geographically coherent meta-population of the wild progenitor.

### Impact of demography and environment on genetic structure

Admixture graphs and residuals from maximum-likelihood analyses were used to model the contribution of migration and gene flow to genetic structure^19^. The best-fitted maximum-likelihood trees reflect established phylogenetic relationships, with *C. echinospermum* basal to a branch containing a single, mostly basal clade of *C. arietinum* and separate branches for each *C. reticulatum* population among which topology mirrors geographic proximity of field sites (Fig. 4). When residuals were used to estimate migration, the increase in proportion of variance explained beyond one migration event was minimal (Supplementary Fig. 3a, b) and at four migration events no residual exceeded 2.7 standard errors. Surprisingly, all predicted migration events implicate gene flow from the relatively distant *C. bijugum* gene pool into *C. reticulatum* populations at Oyalı and Kesentaş sites for which 17.1 ± 1.3% and 11.9 ± 1.6% of their respective ancestries derive from *C. bijugum* (Fig. 4b and Supplementary Table 3). To formally test the hypothesis of admixture, we performed all combinations of four-population tests^20^ and again obtained evidence of gene flow from *C. bijugum* to Oyali (98 out of 105 tests were significantly non-zero) and *C. bijugum* to Kesentaş (43 out of 105 tests) (Supplementary Fig. 4a), with no gene flow detected into other *C. reticulatum* populations (Supplementary Fig. 4b). Similar analyses using the *f*3 statistic suggest that *C. echinospermum* Destek is admixed based on gene flow from both Günaşan (*C. echinospermum*) and Kesentaş (*C. reticulatum*) (*f*3* = *−0.0012; *Z*-score = −4.076). Taken together these analyses indicate a significant contribution from both intra- and interspecific hybridization to the observed genetic structure.

We estimated the potential impact of geography and site ecology on genetic differentiation by analyzing the covariance between allele frequencies among field sites and ecological distance matrices^21^. We find that elevation is more correlated with genome-wide signatures than geographic distance, such that 1 m of elevation is equivalent to 72.4 km of geographical distance (*α*_E_:*α*_D_ ≈ 0.011382) when all wild populations are considered together, or 11.2 km when *C. reticulatum* is considered separately (*α*_E_:*α*_D_ ≈ 0.08933). These values are lower than observed for outcrossing teosinte (*Zea mays*) (*α*_E_:*α*_D_ ≈ 0.153)^21^.

### A demographic model for wild *Cicer*

**Fig. 5:**
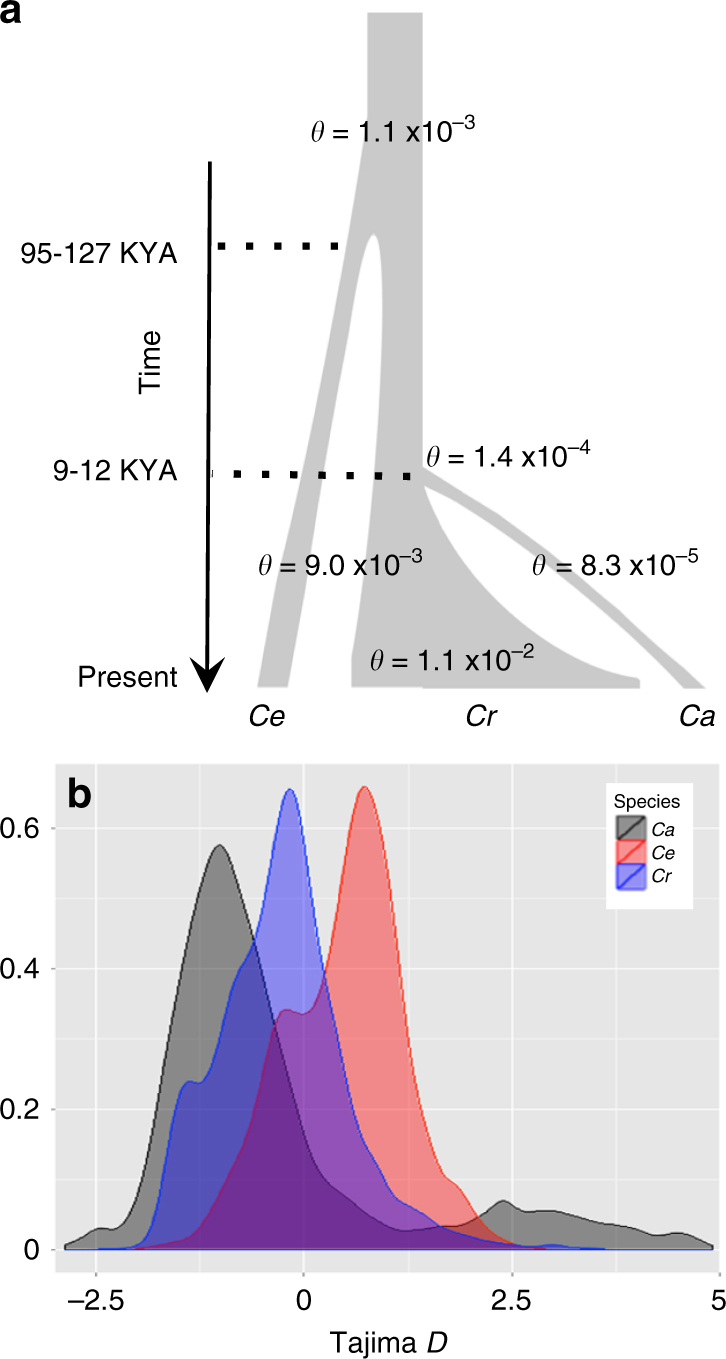
A demographic model and genome-wide patterns of nucleotide variation in wild *Cicer*. **a** Demographic model implemented in G-PhoCS indicating divergence times and theta values based on standing variation present in 16,845 SNPs across each species. **b** Genome-wide estimates of Tajima’s *D* based on 100 bp sliding windows for *C. reticulatum, C. echinospermum*, and cultivated *C. arietinum*

GBS data for a balanced set of 72 accessions (24 accessions from each species, representing all collection sites and populations) were evaluated using a Bayesian inference method^22^ to infer current and ancestral population sizes and population divergence times among the three species (Fig. 5). Bayesian estimates of average nucleotide diversity confirmed the lack of genetic diversity in cultivated chickpea (*θ* = 0.000083 +/−0.000004), orders of magnitude less than either *C. reticulatum* (*θ* = 0.011 +/− 0.003) or *C. echinospermum* (*θ* = 0.0090). These results are in contrast to total counts of polymorphic loci (above) and indicate that both wild species carry similar genetic diversity, in both cases nearly 100-fold greater than the cultivated *C. arietinum*. Archeological evidence, which is extensive in the fertile crescent^23^, suggests a divergence time between *C. arietinum* and C*. reticulatum* at 9–12 K years. Assuming a generation time of 1 year and an average mutation rate of *µ* = 1e^−8^ mutations per site per generation, our models are in agreement with this divergence time (*T* = 10 K years) and estimate the preceding divergence between *C. reticulatum* and *C. echinospermum* at 95–127 K years. Accordingly, the ancestor of the three species had an effective population size (Ne) of 20 K individuals (*θ* = 0.0011) and following divergence the effective population sizes of *C. echinospermum* and *C. reticulatum* increased ~10-fold (Ne = 225 and 275 K, respectively). These same analyses suggest an effective population size for cultivated *C. arietinum* of Ne = 2 K, 100 × less than that of the progenitor *C. reticulatum*, providing further evidence of the magnitude of the domestication bottleneck.

To gain more detailed insight into the genomic consequences of domestication, we compared the full genomes of 26 diverse wild accessions and 7 elite cultivated genotypes sequenced to an average depth of 30–70 × , and 29 breeder-preferred cultivated genotypes previously sequenced^9^. Variant analysis using the ~350 Mbp assembled portion of the chickpea draft genome as a reference revealed 6.97 M high-confidence polymorphic loci (~6 M single-nucleotide polymorphisms (SNPs) and ~1 M insertion/deletion variants). The majority of variants segregate among the 26 wild genotypes (6.7 M), as compared to 451 K polymorphic loci among the 36 cultivated genotypes, including 279 K variants unique to cultivated and potentially derived subsequent to domestication^19^. We estimate that 93.5 to 97.5% of progenitor genetic variation is absent from modern breeding programs, underscoring the reservoir of novel variation captured in these newly archived wild populations. As observed for GBS, *C. reticulatum* populations harbored significantly greater variation compared to *C. echinospermum* (5.3 M compared to 1.7 M polymorphic loci, respectively). In all, 313 K of the total variants occur in deduced protein coding regions, of which 133 K are non-synonymous and of potential functional consequence. Nine-thousand thirty-seven (7%) of these non-synonymous variants segregate within cultivated accessions, among which 5313 are unique to cultivated.

Analysis of Tajima’s *D* in cultivated accessions reveals two tails of values that exceed either wild species. Strong negative values for the majority of the *C. arietinum* genome are consistent with an expanding population that experienced a domestication bottleneck, followed by relaxed selection across much of the genome, while the significant tail of positive values, representing almost 8% of the genome (5.7 MB), indicates diverged loci that might arise through stratification of breeding efforts. Interestingly, several wild populations, in particular Şırnak, Kesentaş, and Güvenli, were found to have Tajima’s *D* values skewed negative as well (Fig. 5 and Supplementary Table 4). Altogether with low values of within-population average pairwise distances (*π* ranged from 9e-^7^ to 2e-^6^), heterozygosity (0.0092 to 0.031) and effective population sizes, these results suggest a scenario in which these populations were recently colonized by a few individuals and are currently undergoing population expansion.

### Seed coat color crypsis suggests wild population adaptation

Implicit in the effort to collect the environmental breadth of chickpea’s wild progenitors are the idealized assumptions that differing environments will harbor locally optimal genotypes, and that some of the corresponding phenotypes will have utility for crop improvement. We provide evidence consistent with this first assumption in the form of seed coat color crypsis, a phenomenon in which seed coat color matches soil color as a means to camouflage progeny from granivores^24–26^. We observed dramatic variation in the coat color of field-collected seed (Fig. 2b), which we subsequently observed to be stable over multiple generations from common garden plants. The color of seed and of soil collected from plant microsites at each field location was imaged and a custom *F*-test used to determine if seed-soil colors were better matched within as opposed to among sites. Considering all within species comparisons, *C. echinospermum* exhibited greater seed-soil color similarity than did *C. reticulatum* (Fig. 2c and Supplementary Fig. 5; custom *F*-test for all species comparisons, DF = 1209; *F* = 284, *P* < 0.0001, Supplementary Table 5). We detected a species-by-native soil effect, with *C. reticulatum* having greater seed-soil color similarity between native versus foreign soils. Seed coat color variation was large among *C. reticulatum* populations, for which we observed both population group and population group-by-native soil effects. At three *C. reticulatum* field sites (Kayatepe, Oyali, and Şirnak) we observed greater similarity between seed coat and native soil color versus foreign soils, suggestive of local adaptation for crypsis. We speculate that these patterns derive from divergent selection imposed by regionally diverse but locally homogeneous soil substrates.

### Wild accessions vary in agronomically relevant traits

Chickpea is a rainfed crop, developing and maturing on residual soil moisture, and during periods of insufficient pre-season rainfall the crop is threatened by terminal drought. Water conservation by crops can ameliorate late-season water deficit^27–31^. Mechanisms to decrease early season soil water use include altered plant architecture (e.g., leaf size and canopy density), differing transpiration responsiveness to moisture availability, and adjustments to phenology, among others.

**Fig. 6:**
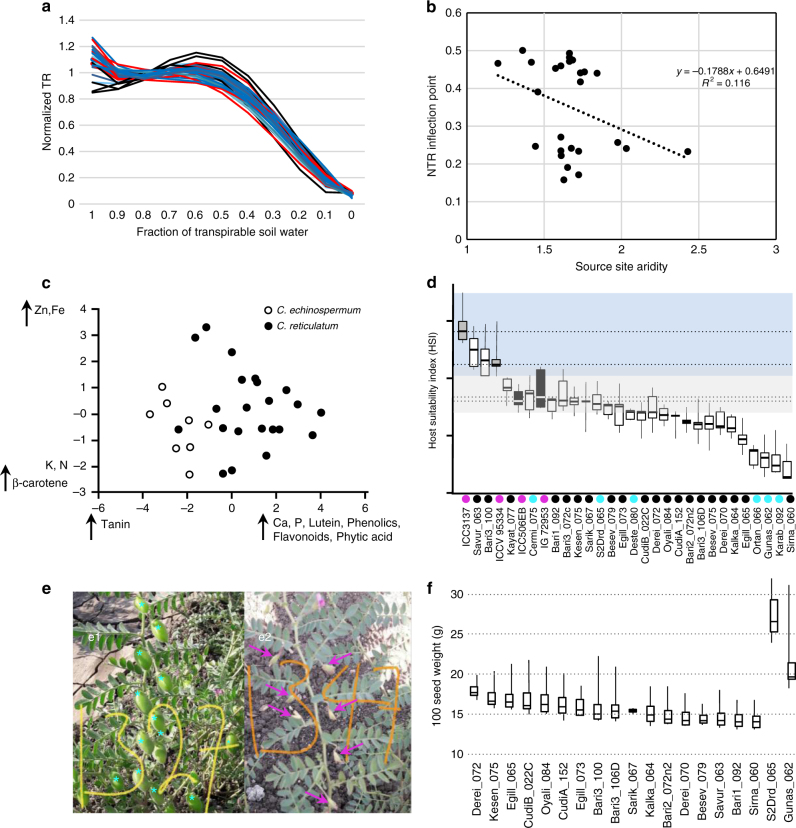
Variation in agronomically important traits in wild *Cicer*. **a** Relationship between the normalized transpiration ratio (NTR) and the fraction of transpirable soil water (FTSW) of twenty-six wild Cicer genotypes and four chickpea cultivars under a scenario of declining soil moisture. Black, cultivated *C. arietinum*; blue, *C. reticulatum*; red, *C. echinospermum*. **b** Inflection point (second derivative of the 4th order polynominal) of the relationship between normalized transpiration rate (NTR) and FTSW against the aridity (precipitation/potential evapotranspiration) of the source site of the 26 wild lines. **c** Principal component analysis of seed nutrient content. **d** Host suitability index for *Helicoverpa armigera* for 26 wild lines and four previously characterized susceptible and resistant accessions from the ICRISAT genebank. Green, tolerant and susceptible checks; red, *C. echinospermum*; blue, *C. reticulatum*. **e** F3 families segregate at high frequency for heat stress tolerance in phenology-normalized lines; typical phenotypes of tolerance (e1)(seed set, blue asterisk) and sensitivity (e2)(flower abortion, red arrows) when grown under high temperatures during March to May in Hyderabad, India. **f** One-hundred seed weight variation among F3 progeny for 19 wild accessions crossed to a recurrent cultivated parent (ICCV96029). Plants were grown in a common field garden. Error bars are standard deviations in panels **d** and **f**

The reproductive cycle of wild *Cicer* populations is punctuated by moisture availability, maturing as soils dry at the onset of increasing summer temperatures. To quantify variation among wild populations for responses to moisture deficit, and to determine if such variation is correlated with home site environments, we characterized transpiration rates (TR) among 26 wild genotypes selected to represent the genetic and source environment diversity of the wild collection, compared to four Turkish elite cultivars (Fig. 6a; Supplementary Fig. 6). Water stress was imposed by moderated decline in soil moisture and TR was analyzed relative to the fraction of transpirable soil water (FTSW); the TR inflection point provides a measure of a genotype’s ability to regulate water loss under soil moisture stress. Wild accessions possessed a wide range of trait values (TR inflection points of 0.41–0.60; Supplementary Table 6), generally exceeding those of the cultivated comparators. Plotting the TR inflection point against source site aridity revealed that genotypes from more arid environments were also more conservative in their water use (Fig. 6b). This relationship was especially evident at the extremes of source site aridity, suggesting that water use strategies are diverged and fixed at extreme sites, with variation and possible balancing selection at intermediate sites. Such a scenario is consistent with, but not proof, of a role in local adaptation.

We also measured TR as a function of diurnal changes to vapor pressure deficit (VPD = atmospheric drought). Residual transpiration not explained by leaf area provided the basis to compare transpiration control among genotypes, for which we observed significant variation (Supplementary Table 7). Deste-080 and Şirnak-060 had high residual TR, with correspondingly high TR under high VPD conditions; such genotypes are potentially adapted to environments with no water limitation. By contrast, Derei-072 and CudiB-022C were among several accessions with highly negative residuals, indicating the capacity of these lines to restrict water loss under high evaporative demand, suggesting their adaptation to water limited environments. We conclude that wild species encompass considerable variation in water use strategies, with several accessions being quantitatively distinct from *C. arietinum* accessions, providing a suite of traits of potential relevance for agricultural improvement.

Especially in the developing world, legume seeds are critical for human nutrition. Following growth of a representative set of wild accessions under common greenhouse conditions, concentrations of six elements, nine amino acids, polyphenolics, and four broad classes of anti-nutritional compounds were evaluated (Supplementary Table 8). Principal components analysis resolved *C. reticulatum* from *C. echinospermum* accessions, with heavy loading of calcium, phosphorus, leutein, total phenolics, flavonoids, and phytic acid on the first PC-axis, and differences in zinc, iron, potassium, total nitrogen, and -carotene as primary factors on the second PC-Axis (Fig. 6c), indicating the potential of wild species for genetic control of nutritional targets.

The pod borer beetle, *Helicoverpa armigera*, is a pervasive agricultural pest of particular concern for chickpea production, even to the extent of crop failure^32^. *H. armigera* larvae were fed either detached leaves or nutritionally complete meal supplemented with lyophilized leaf material, and larval weight gain and survival used to calculate a host suitability index (HSI). HSIs of the same 26 wild accessions used above for drought trials and below for crop introgression, were compared to those of cultivated accessions spanning the range of known susceptibility and tolerance. All but three of the 26 wild accessions had HSI values at least as low as the tolerant checks (Fig. 6d; Supplementary Table 9), including five with HSIs as low as 0.95, which is 3.5 × less than known tolerance in the crop. Standing variation for pod borer resistance in both wild species raises the possibility of distinct alleles or mechanisms, which if true could be used to increase and stabilize pod borer resistance in the crop.

### Towards agricultural innovation

With the long-term objective of breeding with wild alleles, large-scale introgression populations were initiated. Approximately ten-thousand segregating lineages were derived from 26 diverse wild *C. reticulatum* (20) and *C. echinospermum* (6) genotypes crossed into seven crop genotypes that represent major agro-climatic zones of chickpea production (Supplementary Data 1). All parental genotypes were sequenced to at least 30 × coverage, and one full set of 2521 progeny from all 26 wild parents into one early flowering parent was genotyped by sequencing to facilitate trait-marker discovery. To correct for the confounding effect of segregating phenology, we identified a major QTL responsible for flowering time differences between the cultivated ICCV96029 accession and wild genotypes. The corresponding haplotype on chromosome 3 spans 3.55 Mb and includes homologs of the flowering time integrator gene *FT*^33,34^. Marker-based selection for the cultivated haplotype was sufficient to normalize the majority of phenological variation among the respective progeny. With the objective of increasing recombination in the populations, a portion of F2 individuals were intercrossed, yielding ~2000 intercross F2 lineages (iF2s) that are ready to advance as additional recombinant lines.

Phenotypic segregation among F2 individuals and F3/F4 families supports simple genetic control of flowering time^34^, flower color^16^, upright growth^34^, and pod shattering^35^, with more complex segregation of discrete seed coat color classes. In semi-arid climates, including portions of India and Pakistan where chickpea is a vital crop, heat stress is often coincident with drought. In replicated field trials with F3 progeny we observed high rates (249 genotypes among 480 families) of segregation for heat stress tolerance (~40 °C) (Fig. 6e), a phenotype not present in the cultivated parent and that provides an immediate target for introgression breeding. Consistent with our genomic and ecological data, we observed variation for most phenotypes according to the identity of the wild-parent. Even after correcting for the early flowering time locus, maturity date differed by 10 days (13%) between the earliest and latest maturing populations in the ICCV96029 background (Supplementary Fig. 7) demonstrating wild-parent specific influences on phenology; similar wild-parent dependent variation was evident for 100 seed weight (Fig. 6f), as well as aspects of plant architecture, above ground biomass, and seed shape and color. Thus, different wild parents contribute different trait values, which likely reflect different demographic and selection histories, and which we speculate will be of utility for chickpea improvement.

## Discussion

Collections of wild relatives of crops will be most useful to breeding programs if they reflect the breadth of adaptations present in natural populations, which we argue is best accomplished when collections span the full geographic and environmental range of the species. Our collection expands both the genomic diversity and environmental range of the two closest wild relatives of chickpea, increasing the size of the collection by over an order of magnitude.

The variation in substrate, elevation, and climatic range encompassed by the collection increases the likelihood that the assembled germplasm contains variation in phenology, drought, heat and cold stress. Indeed, we observe phenotypes that are correlated with environmental variation in the form of seed color crypsis and responsiveness to drought, and we have identified variation in seed nutrient density, phenology, resistance to pod borer, heat tolerance, and water deficit response. We are also actively exploring segregating variation in *Fusarium* wilt and *Ascochyta* blight resistance, nitrogen fixation and plant architecture, each of which represent traits that are of great interest for chickpea crop improvement.

Our collection also highlights the need for conservation of CWRs. Rapid development in southeastern Anatolia is accompanied by the fragmentation and loss of native landscapes. Two of the populations reported here were lost or fragmented in subsequent years (2014, 2015), while other populations are threatened by human activities. These facts underscore the urgency of the need to collect, characterize, and preserve both in situ and ex situ wild relatives of crops as essential components of humankind’s agricultural heritage and future^1,36,37^.

## Methods

### The collection

For 46 days in May and June of 2013 we surveyed in an area covering over 60,000 sq km and identified 24 sites containing wild *Cicer* populations. We targeted areas with known herbarium collections, available from GBIF (GBIF.org) and Genesys (genesys-pgr.org). New sites were added based on knowledge from local shepherds and targeting similar habitats to known locations. We also made stops on an ad hoc basis.

### Site characterization

We extracted global climatic data based on GPS coordinates for each site from a global climate data set^38^ in DivaGIS following a previously developed approach^39^. To characterize climate at a fine scale, we installed ibutton (Maximintegrated.com) thermochron (ds1921) and hygrochron (ds1923) data loggers to record temperature and humidity just below the soil surface within *Cicer reticulatum* and *C. echinospermum* populations at the end of October 2013. iButtons were placed at five plant microsites within each field site and set to record at 4-h intervals. iButtons were retrieved and data was downloaded in May and October 2014. Data were extracted using a Matlab extraction protocol. Humidity data were normalized following Maxim protocols. Temperature profiles were converted into cumulative degree day estimates following a previously developed procedure^15^ and compared by analysis of variance (ANOVA).

We assessed variation in soil chemistry by analyzing variation in 18 macro and micronutrients and chemical factors. Typically five soil cores, sampling the upper 6 inches of soil, were analyzed at each site and depicted as individual samples in Fig. 2. Analytical chemistry was performed at Dicle University in Turkey following standard protocols. We measured total Cu (mg/kg), total Zn (mg/kg), total Fe (%), total Ca (g/kg), total Mn (g/kg), total Na (g/kg), total K (g/kg), total Mg (g/kg), nitrogen (all forms, as a percentage), organic carbon, inorganic carbon, total carbon, total sulfur (ppm), Lime-CaCO3 (%), pH, EC (µS/cm), phosphorus (P2O5, in mg/kg) and percent organic matter. PCA was conducted to reduce the number of soil chemistry variables that may differ between populations of *C. arietinum*, *C. echinospermum*, and *C. reticulatum*. The variables were either natural logarithm transformed (Zn, Fe, Ca, Mn, Na, Mg, Nitrogen, total carbon, total inorganic carbon, total sulfur, lime-CaCO3, electro conductivity, and potassium), exponential transformed (pH), or not transformed. To test for species differences in principal components, one-way ANOVA was performed on each of the first two principal components. To examine the importance of particular variables, we examined loadings. The highest loadings, above 1.5, are depicted on the graph axes (Fig. 2a).

### Genetic analysis

GBS was used to characterize genetic variation across the wild chickpea accessions. *Hin*dIII and *Nla*III were chosen as two restriction enzymes to digest the genomic DNA and prepare the Illumina libraries. Both restriction enzymes leave a 4-bp overhang, which promotes efficient adapter ligation to insert DNA. Two different types of adapters were used in this protocol. The “barcode” adapter terminates with a 7-bp barcode on the 3′ end of its top strand and a 4-bp overhang on the 5′ end of its bottom strand that is complementary to the end overhang generated by *Hin*dIII. We used a set of 96 barcode adapters, allowing pooling 96 DNA samples into a single lane. The sequences of the two oligonucleotides comprising the barcode adapter are: 5′-ACACTCTTTCCCTACACGACGCTCTTCCGATCTxxxxxxx and 5′-AGCTyyyyyyyAGATCGGAAGAGCGTC2GTGTAGGGAAAGAGTGT, where “xxxxxxx” and “yyyyyyy” denote the barcode and barcode complementary sequences. The second adapter called the “common” adapter was ligated to the overhang end of *Nla*III. The sequences of the two common oligonucleotides were: 5′- AGATCGGAAGAGCGGTTCAGCAGGAATGCCGAG and 5′ CTCGGCATTCCTGCTGAACCGCTCTTCCGATCTCATG. After ligation of the adapters and clean up, fragment size selection was done with AMPureBeads and a PCR step to enrich the library. Fragments were sequenced as 100 bases reads on an Illumina HiSeq4000 at the University of California at Davis Genomics Facility.

Based on field-collected samples, 1385 plant tissue samples were prepared for GBS sequencing across 15 96-well plates. Illumina reads were mapped to the *C. arietinum* CDC Frontier reference^9^ using BWA MEM 0.7.9a-r786^40^. Polymorphisms were called using the GATK pipeline^41^, which considers indel realignment and base quality score recalibration, and calls variants across all samples simultaneously through the HaplotypeCaller program in GATK^41^. Variants were filtered using standard hard filtering parameters according to GATK Best Practices recommendation^42–44^: MQ > 36, QD > 24, MQRankSum < 2. Initial analysis of these results confirms that 19 samples were classified as the wrong species and were subsequently discarded. In addition, since some field samples were from the same plants collected as seed, sibling samples were removed, leaving 1064 unique samples for subsequent analysis. Variant calling of the GBS data identified 148,136 SNPs segregating among the 1064 samples, 25,036 SNPs among the 46 *C. arietinum* samples, 136,638 SNPs among the 656 *C. reticulatum* samples, and 88,976 SNPs among the 306 *C. echinospermum* samples. To circumscribe a smaller set of SNP for population genetic analysis, we considered only loci called in at least 95% of samples. This SNP filtering step and the subsequent analyses were performed within and among the three species. Given differences in polymorphism within species, the final number of polymorphic loci passing this filter step also varied: 11,277 loci for *C. arietinum*, 16,845 loci for *C. reticulatum*, and 21,088 loci for *C. echinospermum*.

Illumina read mapping and variant calling of the WGS data was done using the same BWA MEM and GATK framework, with paired end reads and with adjusted filtering parameters: MQ > 56, QD > 24, MQRankSum < 8, FS < 12, and ReadPosRankSum < 3. These parameters ensure that the reads are mapped to a unique place in the reference with high quality (MQ), that the reads carrying both alleles are comparable in terms of mapping quality (MQRankSum), that the actual variants are called with high quality (QD), and that the variants are not biased towards one strand of the genome (FS) or towards the end of the reads (ReadPosRankSum).

STRUCTURE^17,44,45^ was used to assess admixture and population structure based on 16,845 GBS loci polymorphisms in *C. reticulatum*. The analysis was run for the full set of 1064 accessions and for the three species separately, with the later analyses using a set of loci polymorphic within each species. STRUCTURE was run under the admixture and correlated allele frequency model. Ten independent runs of 10,000 burn-in Markov Chain Monte Carlo (MCMC) iterations followed by 50,000 iterations were performed for 2 to 20 clusters (*K* = 2 to 20). Values of *α* were reported at < 0.1 in all runs at 50,0000 iterations, which is a measure of convergence. Results were inspected using STRUCTURE HARVESTER^46^ and quantified using the Evanno method^18^, which reported log-likelihood values reaching a plateau at *K* = 8 and *K* = 4 groups for *C. reticulatum* and *C. echinospermum*, respectively. Independent runs at the same *K* yielded the same clustering with similar log-likelihood values, and this consistency was taken into account by the Evanno method when assessing clustering. Principal component analysis of the 16,845 GBS loci was performed using the SNPRelate package in R^47^. Agegenet^48,49^ was used to characterize genetic diversity between individuals and between field sites. Because measurements of heterozygosity can be biased by sequencing depth, genomic calls supported by <4 reads were disregarded in calculating heterozygosity. The subsequent estimates of heterozygosity were not correlated with the sample size of the population, mean coverage, or the number of loci identified in an individual accession (Supplementary Fig. 8).

### Population demographic analysis

Demographic analysis was performed using G-PhoCS^22^, a Bayesian inference method that estimates current and ancestral population sizes and population divergence times. Segregating sites among 24 accessions from each of the three *Cicer* species were identified and filtered to remove loci within genic, repeat, or assembled gap regions, similar to other work using the algorithm^22,50^. The set of polymorphisms reduced to 12,508 putatively neutral 100 bp loci. MCMC runs were executed in the same manner, but with different combinations of five parameters, including the initial value of the mutation-scaled population size (theta = default − calculated based on data, 0.0001, 0.001, 0.01), the alpha (*a* = 1, 2) and beta parameters (*b* = 1000, 10,000, 20,000) of the Gamma distribution for the mutation-scaled population sizes and divergence times, and the alpha (*a* = 0.001, 0.01, 0.1) and beta parameters (*b* = 0.00001, 0.0001, 0.001) of the Gamma distribution for the mutation-scaled-migration rates. Each Markov chain was carried out for 1,000,000 iterations and parameter values were sampled after a 100,000-run burn-in period. The program was run using the ‘‘find-finetunes TRUE’’ option to implement a fine-tuning of the parameters automatically during the burn-in period. Diagnostic and trace plots were used to confirm convergence of log-likelihood and model parameters, as well as consistency among all MCMC runs.

All G-PhoCS parameters are scaled by mutation rate and simulations are not dependent on the true value. Effective population sizes are given by *θ* = 4N_e_*µ* and divergence times are given by *τ*** = ***T*µ/g. Translation of parameters to absolute values, divergence times in years and population sizes in number of individuals, was done by assuming an average mutation rate of *µ* = 1e^−8^ mutations per site per generation, and an average generation time of *g* = 1 year. While G-PhoCS does account for difference in population sizes across lineages, we assume this constant mutation rate for all *Cicer* species.

Treemix^19^ was used to construct admixture graphs, fitting phylogenetic trees to the observed variance-covariance matrix of allele frequencies for 24 chickpea populations (15 *C. reticulatum*, 7 *C. echinospermum*, 1 *C. arietinum*, and 1 *C. bijugum*). Each of the populations had between 14 to 83 accessions. Loci with missing data were omitted, resulting in a total of 18,716 SNPs for the analysis. We used blocks of 100 adjacent SNPs. Admixture graphs were first constructed without migration edges using 100 independent replicates to assess the tree with the best likelihood, rooted with the *C. bijugum* population from Kilavuz. Migration edges were sequentially added to this tree using the –se option for generating jackknife estimates and standard errors of the weight attributed to each migration edge. The fitted tree with no migration events explained 97.9% of the variance in ancestry between populations and the tree with one migration event explained 99.8% of the variance (Supplementary Fig. 4C). The addition of further migration edges brought only marginal increases in the variance explained by the fitted model.

*f*3 and *f*4 statistics were calculated for all possible combinations of three and four populations, respectively, from the 24 populations. The Threepop and Fourpop programs provided within Treemix were used to calculate *f*3 and *f*4 statistics and their standard errors in blocks of 100 SNPs. *Z*-scores with absolute value greater than 3 are considered significant^19^. A significantly negative *f*3 statistic from a test of the form *f*3(*PX*; *P*1,*P*2) confirms presence of admixture in a target population *PX* from source populations *P*1 and *P*2. For the *f*4 test, significant non-zero values indicate gene flow in at least one of the pairs of four tested accessions *P*1, *P*2, *P*3, and *P*4. For tests of the form *f*4(*P*1,*P*2;*P*3,*P*4), a significantly positive value indicates gene flow between accessions *P*1 and *P*3 or *P*2 and *P*4 and a significantly negative value indicates gene flow between *P*1 and *P*4 or *P*2 and *P*3^19^.

### Bedassle

The R package BEDASSLE was used to model pairwise genetic distance based on geographical and ecological data. The package relies on a Bayesian approach to model the relative contribution of geographic and ecological pairwise distances to the covariance of allele frequencies^21^. Geological coordinates and altitude values were used to construct the geographic and ecological distance matrices, respectively, and a total of 6466 independent loci called in at least one individual among 21 *C. reticulatum* and *C. echinospermum* populations were used to generate the allele frequency matrix. The beta binomial model was used to account for over-dispersion. Three independent estimations ran for 5 million generations each. MCMC marginal traces were visualized removing 25% for burn-in to assure parameter convergence.

### Seed color variation

To assess variation in color among seeds and their source soils, we followed a previously developed protocol^26^. Seeds from plants grown in a greenhouse were used to minimize parental environmental effects on color, which we also determined to be similar to the seed obtained from the field-collected parents. Images of each of five seeds per plant accession were captured using a dissecting microscope (Olympus, Model# SZX16; Olympus, Center Valley, PA, USA). Soils collected from the source habitats at the same time as seed collection were also imaged using the same dissecting microscope. From 17 *Cicer* field sites, five independent soil samples were analyzed. Prior to their analysis, soil samples were dried at 30 °C for 2 weeks, followed by division into three aliquots spread over ~ 1 cm^2^ area for imaging.

Photo-microscopy was standardized using the following set-up: 3, 30-watt, 110 V fluorescent light boxes were used to illuminate samples from three angles, reducing shadow effects to the extent possible. A consistent exposure time and focal distance was set using the X-Rite Mini^TM^ ColorChecker color standards, with color pixels for each of the images maintained at <245 of the possible 255 in the red, green and blue (RGB) color channels. Each session involved imaging six gray reflectance standards, 19 to 24, from the X-Rite ColorChecker. The six standards reflect known amounts of light equally at all wavelengths, enabling downstream adjustments in the image processing. Each image was saved in a Tagged Image File Format (TIFF) format.

ImageJ software was used in the digital quantification of color^51^. After separating images from their neutral backgrounds, average pixel values of RGB color channels were recorded. Gray standards were similarly treated, without the need to remove the neutral background.

Potential artifacts introduced by the imaging system were removed by standardization, following well established protocols^26,52^. The non-linear response of the camera’s RGB color sensors to increasing radiance was corrected by linearizing the raw RGB values to true values of the six gray standards. These values were further equalized according to the six gray color standards. Both linearization and equalization steps were achieved using standard equations, yielding values in the three parts of the spectrum: Long Wave, Medium Wave, and Short Wave^52^. Red, green, and blue color measurements were recorded based on the mean of five measurements per seed and three measurements per soil. These means per line and soil sample were used in the following analyses to test whether seed and soil samples were more similar in color when compared to their native soil than foreign soils.

To directly test whether seeds and soils from each site are more closely associated in color, site mean values for seeds and soils were calculated for all three colors. These means were then used to quantify Euclidean distance in three dimensions of each seed sample to its native and all foreign site soil samples. A mixed-model ANOVA was performed to specifically test the hypothesis that seed color is more similar to their native site’s soil than between all other site’s soils. We performed a similar analysis using populations assigned by STRUCTURE from SNP variation. Euclidean distance values were square-root transformed to meet assumptions of normality and homoscedasticity of ANOVA. Because of the repeated-measures of Euclidean distance per genotype, all mixed-model ANOVAs with plant genotype nested within-population were treated as a random effect, thus accounting for the non-independence of Euclidean distance measurements for population and soil comparison estimates.

### QTL analysis of days-to-flowering and of growth habit

Plants of the recombinant inbred population CRIL2^35^ derived from an interspecific cross between *C. arietinum* genotype ICC 4958 × *C. reticulatum* genotype PI 489777 were assayed. Scarified seed of 107 RILs were planted into 7 cm diameter wide conetainers filled with autoclaved artificial soil. Plants were grown in a glasshouse with supplemental lighting to a total of 14 h of daylight per day. Containers were watered every 2–3 days, and fertilized at weekly intervals with a dilute fertilizer mix lacking in nitrogen but containing all other macro and micronutrients. Days to first flower was recorded when the first bud reached full anthesis for each of 3–5 individual plants of each RIL. Plants that did not exhibit flowering by 10 weeks after planting were noted as late-flowering genotypes. For detection of QTLs, the average days-to-flowering for each RIL was used in analysis with QTL-cartographer, in conjunction with 2956 molecular markers (SNPs) obtained from GBS of the RILs and their parental genotypes. QTL detection threshold was set to LOD score of 3.0 and the detected QTL LOD score at the FT locus exceed 20.0.

### Comparing water use in wild accessions

We selected 26 wild accessions that are parents of introgression lines and four cultivated lines for common garden studies to assess differences in transpirational control in response to high VPD (high vapor pressure deficit) and limited soil water availability. In the first experiment we exposed plants to high VPD. In the second, we exposed plants to dry-down conditions simulating the terminal drought typically experienced by chickpea in water limited production zones (such as South Asia and East Africa, as well as most areas where chickpea is grown on residual soil moisture after a rainy season).

The VPD experiment was conducted during the spring season (18 March to 21–22 April 2016) at the Dicle University glasshouse. Plants were grown in 6'' pots (top diameter 18.5 cm, height 15.5 cm, bottom diameter 15.5 cm) filled with soil. Four seeds of each genotype were sown in each pot, irrigated with 500 ml of water immediately after sowing and twice on alternate days with 250 ml until the seedlings emerged uniformly. Plants were thinned to two individuals per pot at 15 day after sowing (DAS). The experimental design was a complete randomized block design with six replicates of each genotype. The experiment was carried out over 2 days and 3 replications per genotype were tested on each of the days, on 21 April 2016 and 22 April 2016 (so-called VPD1, and VPD2), respectively. Data for the first three replicates were recorded on 21 April 2016, i.e., 32 DAS. The afternoon before starting VPD measurements, pots were watered to saturation and allowed to drain overnight, which equilibrates the plant and soil water potentials. This treatment allows the plant water status to reach equilibrium with the soil water status such that the difference between the soil water potential and the stem/leaf water potential is as close as possible to zero. The following morning (7:00–8:00 a.m.), the soil surface was covered with a plastic sheet and a uniformly thick layer (3–4 cm) of plastic beads to reduce soil evaporation. Transpirational water loss was determined by weighing pots at seven time points throughout the day under the prevailing temperature and RH % conditions, when VPD increased through the time course of the day. VPD was calculated from these temperature and relative humidity % data using the formula:

$${\mathrm{VPD}} = \left( {\left( {100 - {\mathrm{RH}}} \right)/100^ \ast {\mathrm{SVP}},\,{\mathrm{SVP}}\,\left( {{\mathrm{Pascals}}} \right) = 610.7^ \ast 107.5{{T}}/\left( {237.3 + {{T}}} \right)} \right.$$

To ensure uniformity of plant stature, a total of 600 pots were initially planted and only those containing plants of similar size among the replicates of a same genotype were selected for the VPD and dry-down (below) experiments, consisting of 360 pots for dry-down and 180 pots for VPD.

### Transpiration and leaf area assessments

Calculations of transpiration rate (TR, *g* water transpired per unit of leaf area) required acquiring both transpiration data (*T*) and leaf area (LA) data to compute TR = *T*/LA. All *T* data obtained from the VPD experiment consisted in pot weight losses from consecutive weighings. Transpiration was calculated both per hour and for the entire experimental time from 8:00 a.m. to 4:00 p.m. (for the VPD response), and for daily values (for the dry-down experiment). Before measurement all pots were fully saturated with water and left to drain excess water overnight to reach field capacity. For example for each hour transpiration, *T* = (first weight (8:00 a.m.)− second weight (9:00 a.m.)), daily *T* = total transpiration (from 8:00 a.m. to 4:00 p.m.), day *T* = weight day_*n*_−weight day_*n*+1_.

Leaf area was measured by digital scanning and the WinFolia program. All leaves were separated from the shoots and placed on the scanner glass for analysis. The results were measured as cm^2^ pot^−1^.

$${\mathrm{TR}} = \left( {{\mathrm{Transpiration}}/{\mathrm{Leaf}}\,{\mathrm{area}}^ \ast 1000} \right)/60\left( {{\mathrm{expressed}}\,{\mathrm{in}}\,{\mathrm{mg}}\,{\mathrm{cm}}^2{\mathrm{min}}^{ - 1}} \right)$$

There was a close relationship between the transpiration *T* and the leaf area (the larger the canopy the larger the transpiration). Residual transpiration that was not explained by the leaf area was calculated as:

$${\mathrm{Residual}}\,{{T}} = {\mathrm{Total}}\,{\mathrm{transpiration}} - \left( {a^ \ast {\mathrm{leaf}}\,{\mathrm{area}} + {{b}}} \right)$$

where *a* and *b* are the slope and intercepts of the regression linear equation between total transpiration and leaf area. The purpose of calculating these residual was to investigate how much of these residual transpiration could be explained by TR differences, and especially TR differences under high VPD.

### Dry-down drought stress

The dry-down experiment was conducted 18 March to 21 May 2016 at the Dicle University glasshouse. Plants were grown in 6'' pots filled with soil. Four seeds of each genotype were sown in each pot on 18 March 2016. Five-hundred milliliter of water was applied immediately after sowing and twice on alternate days with 250 ml until the seedlings emerged uniformly. Plants were thinned to two individuals per pot at 15 DAS. The experimental design was a complete randomized block design with two water treatments (well-watered (WW) and water stressed (WS)) as the main factors and genotypes as the sub-factors, with six replicates for per treatment. Plants were maintained with full irrigation before beginning the dry-down phase of the experiment. The afternoon before the start of the dry-down, soil was fully saturated and the pots were allowed to drain overnight. The following morning (5:30–06:00 a.m.), the soil surface of each pot was covered with a plastic sheet and a uniformly thick layer (3–4 cm) of plastic beads was applied to reduce soil evaporation. Initial pot weight was considered to be the field capacity weight. On subsequent days, pots were weighed at 6:00 to 7:00 a.m., and were supplemented with water to create the WW and WS treatments. Transpiration on each day was calculated as the difference in water loss between successive days plus water added to pot between the two successive weighings. WW plants were maintained at 80% field capacity, which was 200 g below the field capacity of a typical 6'' pot, meaning that water losses in excess of 200 g per pot were added back to the pots on a daily basis. WS was imposed by partial compensation of the daily water losses from plant transpiration. Typically plants were allowed to lose 50 g/day at the beginning of the drydown, then 70 g/day at the end of the drydown, with water loss in excess of these threshold values added back to the pots on a daily basis. Thus, pots lost similar quantities of soil water each day, irrespective of differing transpiration rates, and were thus exposed to similar kinetics of water stress imposition. The experiment was terminated when transpiration of WS plants was <10% (0.1 normalized transpiration ratio (NTR) value; described below) of WW plants. Harvested plants were dried in a 60 °C oven for 48 h, and dry weight measurements recorded. At the end of the transpiration measurements, fraction of transpirable soil water (FTSW) per day was calculated using the morning pot weight measurements and calculated as: FTSW = (Pot weight day *n* - Final pot weight)/(Initial pot weight-Final pot weight). Values represent the fraction of water that was available for daily transpiration and hence are an indication of the level of stress experienced by the plant^53^. Data were analyzed using SAS Software (SAS Institute Inc., 1998, SAS/STAT user’s guide, Version8, Cary, NC). Each daily NTR was plotted against the corresponding FTSW using a plateau regression method^54,55^.

### Seed nutrient analysis

Variation in selected amino acids, minerals, polyphenols, and anti-nutritional content among seeds were determined by gas chromatography (GC)-mass spectrometry (MS), liquid chromatography (LC)–MS, and atomic absorption spectroscopy (AA) on two replicate seeds of 21 *C. reticulatum* and eight *C. echinospermum* genotypes grown in a common garden greenhouse under 16 h days at the University of Saskatchewan on Sunshine Mix #4 (Sungrow, Agawam, MA, USA). Thirteen amino acids (alanine, glycine, serine, proline, hydroxyproline, phenylalanine, valine, leucine, isoleucine, threonine, methionine, lysine, and tryptophan) were quantified by GC-MS. Zn, Fe, Ca, and K were analyzed by AA. P and N were determined by micro-Kjeldahl (acidic) digestion, followed by colorimetric (flow-injection) analysis. The carotenoid lutein (ng/mg) and the pigment carotene (ng/mg) and four anti-nutrients [Tannin (gCATE/g), phenol (gGAE/g), Flavonoid (gCATE/g), and phytic acid (mg/g)] were measured by LC–MS.

To test for species differences in seed content, means were calculated per genotype and the subsequent genotype means were used in the analyses. Because of the non-normal distribution of the data, even after transformations, we performed analysis using a generalized linear model with a single factor (i.e., species) using a Poisson distribution unless otherwise stated. Analyses for hydroxyproline, phenylalanine, methionine, and tryptophan were performed using a normal distribution for non-transformed data. Prior to testing for species effects on each element or compound, we performed a multivariate analysis of variance (MANOVA) testing for a species effect on the four anti-nutrients. Principal component analyses were performed on the amino acids, six seed elements (zinc, iron, calcium, potassium, phosphorus, nitrogen), four broad classes of anti-nutritional compounds (tannin, total phenolics, total flavonoids, phytic acid), carotenoid lutein and pigment carotene.

To assess variation in seed mineral content in more depth, we used induced-coupled plasma mass spectrometry (ICPMS) following well established protocols^56,57^. We used 218 genotypes grown in University of California (UC) Davis potting mix at UC Davis under 16 h days, and 26 genotypes (20* C. reticulatum* and 6 *C. echinospermum*) grown on Sunshine Mix #4 (Sungrow, Agawam, MA, USA) at the University of Saskatchewan. Five replicate seeds of each genotype were analyzed for 20 different nutrients: boron, sodium, magnesium, aluminum, phosphorus, sulfur, potassium, calcium, manganese, iron, cobalt, nickel, copper, zinc, arsenic, selenium, rubidium, strontium, molybdenum, cadmium. For each genotype, nutrients were measured on three to five samples. Prior to statistical analyses, means per genotype were calculated for each element and all analyses were conducted using genotype means.

To test whether the two species (*C. reticulatum* and *C. echinospermum*) differed in seed elemental content, we performed a multivariate analysis of variance (MANOVA- PROC GLM) on species that included all 20 nutrients (Supplementary Table 8). Wilks’ Lambda and Pillai’s Trace test statistics were calculated for MANOVA analysis and because they consistently had common results only Wilks’ Lambda results are shown. One-way ANOVA’s (PROC GLM) were performed for each element to test for species differences for each nutrient. To help meet assumptions of MANOVA and ANOVA, the following data were natural logarithm transformed: boron, sodium, aluminum, phosphorus, manganese, copper, selenium, rubidium, molybdenum, cadmium; were square-root transformed: cobalt, nickel, arsenic; or were not transformed: magnesium, sulfur, potassium, calcium, iron, zinc, strontium. To test for species differences, we performed LSMEANS comparisons and to directly test whether cultivated genotypes differed from wild-type, we performed linear contrasts. To reduce the number of elemental factors analyzed, we performed principal component analyses (PROC PRINCOMP) on the 20 elements. The principal component analysis was followed by MANOVA and ANOVA on the principal axes to identify species differences.

### Assessment of pod borer host suitability

We used a detached leaf assay, following established methods^58^, to assess pod borer (*Helicoverpa armigera*) weight gain, survival, and damage to the host plant under controlled conditions. Chickpea plants of 26 wild lines (the same whole genome sequences lines above), along with a known resistant wild line (IG72952), a resistant cultivated (ICC506EB) and two susceptible cultivated lines (ICC3137 and ICCV 95334) were grown under greenhouse conditions. At 30 and 60 days after seedling emergence, terminal branches (2 to 3 fully expanded leaves and a bud) were bio-assayed for growth and survival impacts on neonate larvae of *H. armigera* using the detached leaf assay. The chickpea branches were cut with scissors and immediately planted in a slanting manner in 3% agar–agar medium in a 10 cm diameter plastic cup (250 ml capacity). There were five replications for each accession in a completely randomized design. Ten neonate larvae of *H. armigera* raised in the laboratory were released on the chickpea leaves with a camel hairbrush. The cups were kept in the laboratory at 27 ± 2 °C and 45 to 65% RH. Observations were recorded at 6 days after initiating the experiment (when the differences between the test genotypes became most apparent) for branches bio-assayed at 30 days after seedling emergence, and at 5 days after initiating the experiment at the reproductive stage^58^. Host suitability was calculated as HSI = ((larval weight gain)/(leaf damage rating))*(larval survival). Comparisons among all genotypes for HSI were done using Kruskal–Wallis H test with Dunn’s post hoc test (with Bonferroni *p* adjustment to adjust for multiple comparisons).

### Construction and phenotyping of introgression populations

We created wild introgression populations to establish a resource for association mapping of climate-resilience traits and to initiate breeding with wild alleles. For maternal parents we used elite varieties that are farmer-preferred and adapted to the major chickpea growing regions of the world, in Turkey (the Middle East), Canada (north America), Ethiopia (eastern Africa), India (south Asia), and Australia (Australasia, on-going and not described here) (Supplementary Data 1) thereby maximizing potential impact on global chickpea production. Each of these cultivated regions has distinct seasonalities and accompanying biotic and abiotic factors to which both historical selection and modern breeding programs have adapted the crop. *C. arietinum* is grown during the spring and early summer in Turkey; during the summer in Canada; during the post monsoon fall in Ethiopia; in the winter in India; and as a fall-winter crop in Australia.

Wild parents were selected to maximize genetic diversity and the variety of source climates. Initially, one accession was selected from each field site, which covered all STRUCTURE-defined populations (Fig. 4). Four *C. reticulatum* sites with the greatest range of pairwise nucleotide diversity (Baristepe3, Beşevler, Dereiçi, and Egil) were represented multiple times among the wild parents, selecting samples within these sites with the greatest differentiation to maximize allelic diversity. Practical considerations of seed availability and differential success in crossing, especially for certain *C. echinospermum* accessions^59^, further refined the set of parents (Supplementary Data 1).

For the subset of populations derived from cultivated parent ICCV96029, and which consequently segregate for a cultivated early flowering locus (a candidate FT locus derived from ICCV96029 and described here), we genotyped progeny using KASP^60^ for the FT locus. Molecular phenotyping for flowering time provided a covariate for phenotyping studies in which phenology complicates analysis (for example in heat tolerance analysis). The same marker provides the basis for rationale selection of population subsets for specific phenotyping tasks. Subsequent genotyping (GBS) of the full set of ICCV96029-derived progeny permits “reverse-introgression” of cultivated haplotypes for any given trait tagged with a molecular marker. Here, we describe the utility of a flowering time marker, but a similar approach was used to reduce branching based on a marker linked to branching frequency that we identified on chromosome 1. We anticipate using the same logic for other domestication-related traits, such as pod shattering. The concept of adjusting phenotypes with known molecular markers is not novel, nor are candidate genes that we implicate. However, the ability to “reset” the genome of a crop wild progenitor, or of derived segregating populations, at a few key domestication loci is likely to be especially powerful. We envision creating lineages bearing key cultivated traits, but that otherwise possess high levels of wild variation. We argue that a collection of such lineages will provide an ideal system in which to test the agronomic utility of the wild backgrounds. Such an approach could be especially powerful in the case of traits conferred by multiple loci of small effect size, for example, removing deleterious mutations that are predicted to accumulate during domestication and breeding.

Here, we report primarily on crosses using the early flowering Indian parent ICCV96029 as the maternal parent, although populations have been developed using a wider range of elite cultivated parents (as described above and in Supplementary Data 1). The majority of F2 seed were advanced to F3 seed in cultivated fields. An additional ten to fifteen F2 plants from each population were grown in a greenhouse for intercrossing, yielding ~2000 intercrossed F2 lines that have yet to be advanced further; intercrossing was intended to increase recombination and thus genetic power without the need for correspondingly larger populations.

Population development is an ongoing activity. For example, all ICCV96029-derived progeny are at the F4 stage or beyond. To conserve genetic diversity within each F2-derived lineage, we typically sow and collect seed from 10 to 20 siblings of each familial generation. Although we aim to select individual plants from each F2-derived lineage for development of pure-line recombinant inbred lines, our focus to date has been to screen multiple individuals per F2-derived lineage, which has the advantage of maintaining variation.

F2 progeny were screened for phenology, biomass, growth form, sterility, yield and pod shattering, while F3 seed was characterized for seed coat color and seed weight, both of which are maternally controlled traits and thus indicative of the F2 genotype. Plants were harvested individually as they attained maturity. For plants with shattering pods, seed was collected continuously from individual plants until maturity. Maturity date represents the harvest date for individual plants relative to the date of germination. Plant architecture was estimated from four values: a simple constrained text based description of growth pattern (upright, intermediate or prostrate; highly branching or not), and measurements of plant height, the widest point and then the axis perpendicular to the widest point. Volume was approximated assuming the shape of an ellipse: (4/3) × (*a* × *b* × *c*). Density was estimated as the ratio of biomass to volume. At harvest, the above ground portion of individual plants was collected by cutting the plant at soil level. Plants were dried in heavy duty brown paper bags: at least 1 week in the field, 2 weeks in a warm greenhouse with continuous air movement and no cooling, and 1 week in a temperature controlled (60 °C) drying room. Shattering was calculated as the ratio of mature seed released from or remaining within pods after drying.

### Field trials for heat stress

A total of 480 F3 families (473 F3 families and seven heat tolerant and sensitive cultivated genotypes) were screened under field conditions during March to May 2017. To reduce the impact of phenology, the 480 families were genotyped for the chromosome 3 early flowering FT-linked locus (as described in the main text) and selected to equally represent early flowering homozygotes and heterozygotes. The experiment was conducted using an alpha-lattice design with two replications, with each replicated consisting of ~7 plants per family. Based on a visual score of flower abortion and the number of filled pods per plant, 249 genotypes were scored as tolerant to heat stress.

### Data availability

All sequencing data can be found in the NCBI Umbrella BioProject PRJNA353637, which includes Illumina data from GBS sequencing of over 1000 wild *Cicer* accessions (PRJNA416006) and from WGS sequencing of 224 wild *Cicer* accessions (PRJNA416007).

Ecological, soil chemistry, and phenotypic data are available from the dryad repository, 10.5061/dryad.74699. We declare that all other data supporting the findings of this study are included in the manuscript and Supplementary Information files or is available from the corresponding author upon request.

## Electronic supplementary material

Supplementary Information

Description of Additional Supplementary Files

Supplementary Data 1

Supplementary Data 2
