# Supplementary material for: Ecology and genomics of an important crop wild relative as a prelude to agricultural innovation
Source: Nat Commun. 2018 Feb 13;9:649. doi: 10.1038/s41467-018-02867-z (PMC5811434; doi:10.1038/s41467-018-02867-z)
Supplement: Supplementary file 1 — Supplementary Information [file 41467_2018_2867_MOESM1_ESM.pdf]

Supplementary Table 1. ANOVA of differences among sites in heating degree days, following the Chen et al (2012) mode both assuming either no vernalization or vernalization.

Model without vernalization

Source	SS	df	MS	F	Prob>F
**Thermal Units**	5746.2	16	359.139	26.77	$4.16e^{-80}$
**Error**	247468.6	18445	13.417		
**Total**	253214.8	18461			

Model with vernalization

Source	SS	df	MS	F	Prob>F
**Thermal Units**	2653.5	16	165.841	6.62	$3.02e^{-15}$
**Error**	462193.3	18445	25.058		
**Total**	464846.8	18461			

SS, sum of squared
df, degrees of freedom
MS, mean squared
F, F statistic
Prob>F, probability value associated with F.

Supplementary Table 2. Summary statistics on samples sequenced and polymorphisms.

	**All**	***Cicer arietinum***	***Cicer reticulat***	***Cicer echinospermum***
**Number of Samples Sequenced**	1366*	124	774	342
**Number of Samples Retained**	1064**	56	656	306
**Number of loci polymorphic within each set**	148,136	25,036	136,638	88,976
**Number of loci polymorphic within each set for population genetic analysis**	16,845	11,277	16,845	21,088

*Includes 126 *Cicer bijugum* samples
**Includes 46 *Cicer bijugum* samples

Supplementary Table 3. Admixture proportions for up to 4 migration events in the fitted accession tree (Figure 4b)

**Migration event proportion**	**Admixture ± standard error**	***P*-value**
Kilavuz!Oyali	17.1±1.3%	<2.23e^{-308}
Kilavuz!Kesentas	11.9±1.6%	6.19e^{-13}
Kilavuz!(Sirnak,CudiA,CudiB)	6.3±1.0%	1.39e^{-10}
Kilavuz!Bari2	3.8±0.01%	4.47e^{-09}

P-value, probability value.

Supplementary Table 4. Population genetic statistics across 16,845 SNPs.

Population	Species	Hs	Tajima D	PI
Cultivated	*arietinum*	0.08927	-0.757653888	$2.00e^{-06}$
Baristepe 1	*reticulatum*	0.03978	-0.486651002	$4.00e^{-06}$
Baristepe 2	*reticulatum*	0.0979	-0.018507921	$5.13e^{-06}$
Baristepe 3	*reticulatum*	0.0968	-0.102281894	$5.06e^{-06}$
Besevler	*reticulatum*	0.09368	-0.071017043	$6.48e^{-06}$
Cudi A	*reticulatum*	0.07654	-0.030666944	$5.30e^{-06}$
Cudi B	*reticulatum*	0.05777	0.085340858	$6.26e^{-06}$
Dereci	*reticulatum*	0.08622	0.055901881	$6.37e^{-06}$
Egil	*reticulatum*	0.09374	-0.113811878	$6.22e^{-06}$
Kalkan	*reticulatum*	0.08514	0.074683336	$7.71e^{-06}$
Kayatepe	*reticulatum*	0.08221	-0.221334851	$5.18e^{-06}$
Kesentas	*reticulatum*	0.02709	-0.811521326	$2.29e^{-06}$
Oyali	*reticulatum*	0.07254	-0.187121316	$4.86e^{-06}$
Sarikaya	*reticulatum*	0.07249	-0.321330115	$5.57e^{-06}$
Savur	*reticulatum*	0.07954	-0.100459006	$7.02e^{-06}$
Sirnak	*reticulatum*	0.00924	-1.295944461	$9.34e^{-07}$
Cermik	*echinospermum*	0.0955	-0.140202886	$5.31e^{-06}$
Destek	*echinospermum*	0.10344	-0.170023083	$2.82e^{-06}$
Guvenli	*echinospermum*	0.03137	-0.915508952	$2.21e^{-06}$
Gunasan	*echinospermum*	0.08255	0.296570175	$6.86e^{-06}$
Karabachi	*echinospermum*	0.10629	-0.278990661	$4.12e^{-06}$
S2Drd	*echinospermum*	0.09404	0.080038862	$6.54e^{-06}$
Ortanca	*echinospermum*	0.08558	-0.365004572	$3.77e^{-06}$

Hs, average gene diversity.
PI, pairwise nucleotide diversity.

Supplementary Table 5a. Results of mixed-model ANOVA on Euclidean distance from seed-soil color comparing soil category (i.e., Native vs. Foreign), species (Species), and population nested within species (Population). Genotype was treated as a random effect.

Source	Df	F	P-value
Soil Category	1, 209	0.45	0.5046
Species	2, 209	283.97	<0.0001
Soil Category X Species	2, 209	2.71	0.0686
Population	16, 209	11.75	<0.0001
Soil Category X Population	16, 209	3.76	<0.0001

Supplementary Table 5b. Results of one-way ANOVAs performed for each population testing whether seed-soil color distances for a given population differs from soil from different populations of the same species.

Seed Population	df	F	P-value
**Ech populations**			
Cermik	4, 20	1.12	0.3757
Gunasan	4, 12	27.27	<0.0001
Karabace	4, 48	26.00	<0.0001
Ortanca	4, 8	11.05	0.0024
**Ret populations**			
Bari1	13, 104	9.53	<0.0001
Bari2	13, 91	1.80	0.0554
Bari3	13, 247	18.08	<0.0001
Besev	13, 91	2.00	0.0296
Cudi	13, 455	38.91	<0.0001
Derici	13, 117	1.99	0.0275
Egil	13, 78	1.25	0.2619
Kalkan	13, 65	8.22	<0.0001
Kayatepe	13, 78	9.78	<0.0001
Kesentas	13, 130	11.88	<0.0001
Oyali	13, 130	5.11	<0.0001
Sarikaya	13, 104	10.99	<0.0001
Savur	13, 13	73.35	<0.0001
Sirnak	13, 442	57.57	<0.0001

df, degrees of freedom.
Custom F-test; F, F value.
P-value, probability value

Supplementary Table 6. Fraction of transpirable soil water (FTWS) thresholds of wild and cultivated *Cicer* accessions. FTSW threshold is the inflection point at transpiration declines upon exposure to progressive soil water deficit.

Genotype	FTSW threshold	Approximate SE	Approximate %95	Confidence Limits
BARİ 1 092	0.425	0.008	0.407	0.443
BARİ2 072N2	0.541	0.013	0.514	0.567
BARİ3 072C	0.538	0.014	0.510	0.567
BARİ3 100	0.572	0.013	0.546	0.599
BARİ3 106D	0.530	0.009	0.512	0.548
BESEV 075	0.495	0.011	0.473	0.518
BESEV 079	0.461	0.009	0.441	0.480
ÇAĞATAY	0.494	0.012	0.469	0.52
ÇERMİK 075	0.496	0.010	0.476	0.517
CUDİA 152	0.564	0.012	0.539	0.590
CUDİB 022C	0.547	0.013	0.520	0.573
DEREİ 070	0.504	0.010	0.482	0.525
DEREİ 072	0.505	0.013	0.477	0.532
DESTE 080	0.430	0.013	0.402	0.457
DİKBAŞ	0.558	0.014	0.529	0.587
DİYAR 95	0.409	0.020	0.368	0.449
EĞİL 065	0.582	0.009	0.563	0.601
EĞİL 073	0.554	0.010	0.530	0.578
GÖKÇE	0.449	0.013	0.422	0.476
GUNAS 062	0.469	0.014	0.441	0.496
KALKA 064	0.496	0.011	0.473	0.518
KARAB 092	0.601	0.015	0.571	0.630
KAYAT 077	0.577	0.010	0.556	0.597
KESEN 075	0.480	0.011	0.456	0.503
ORTAN 066	0.553	0.017	0.519	0.586
OYALİ 084	0.502	0.009	0.483	0.522
S2DRD 065	0.470	0.014	0.442	0.498
SARİK 067	0.498	0.010	0.476	0.519
SAVUR 063	0.467	0.012	0.442	0.492
SİRNAK 060	0.533	0.009	0.513	0.553

SE: standard error of the mean.

Supplementary Table 7. Transpiration rate (TR) and residual TR values of genotypes at high VPD conditions.

	VPD1		VPD2	
**Genotype**	**TR**	**Residual TR**	**TR**	**Residual TR**
BARİ1-092	0.623	1.249	0.603	1.355
BARİ2-072N2	0.474	7.916	0.504	-0.725
BARİ3-072C	0.588	5.808	0.528	-2.468
BARİ3-100	0.471	1.584	0.543	4.111
BARİ3-106D	0.429	-3.496	0.509	-2.618
BESEV-075	0.502	3.835	0.632	5.401
BESEV-079	0.498	1.300	0.540	-0.218
CERMİK-075	0.552	1.649	0.626	4.899
CUDİA-152	0.513	-0.489	0.507	-0.754
CudiB-022C	0.386	-2.432	0.459	-2.503
ÇAĞATAY	0.480	-0.396	0.476	2.472
DEREİ-070	0.455	-2.566	0.486	-2.760
DEREİ-072	0.334	-8.968	0.458	-2.047
DESTE-080	0.991	6.766	0.799	0.899
DİKBAŞ	0.498	-2.606	0.532	-5.526
DİYAR-95	0.607	5.904	0.721	15.325
EGİL-065	0.448	-0.368	0.504	-0.057
EGİL-073	0.468	-4.435	0.613	3.960
GÖKÇE	0.412	-1.416	0.317	-10.799
GUNAS-062	0.549	-3.516	0.545	-3.835
KALKA-064	0.844	2.354	0.471	-2.428
KARAB-092	0.649	8.592	0.503	-0.794
KAYAT-077	0.463	2.857	0.524	-2.098
KESEN-075	0.367	-6.292	0.565	0.245
ORTAN-066	0.462	-3.060	0.786	4.242
OYALİ-084	0.491	-0.170	0.547	0.507
S2DRD-065	0.477	-5.786	0.497	-5.383
SARİK-067	0.486	-1.755	0.522	-0.249
SAVUR-063	0.518	-2.507	0.703	0.359
SİRNAK-060	1.101	0.131	0.789	1.683

VPD, vapor pressure deficit.

Supplementary Table 8a. MANOVA of Mineral Nutrient content in seeds. Wilks' Lambda and Pillai's Trace test statistics from MANOVAs using all 20 elements (All data) and the 16 elements (i.e., without Boron, Aluminum, Arsenic, and Selenium; Subset) testing species and population/origin nested within species effects. Separate analyses were performed for population and origin effects.

Source	df	Statistic value	F-Value	P-value
BASED ON POPULATION				
All data				
Species				
Wilks' Lambda	40, 506	0.2316	13.63	<0.0001
Pillai's Trace	40, 508	0.9562	11.63	<0.0001
Population(Species)				
Wilks' Lambda	200, 2296.5	0.0693	3.97	<0.0001
Pillai's Trace	200, 2620	2.1743	3.64	<0.0001
Subset 16 elements				
Species				
Wilks' Lambda	32, 514	0.3775	10.08	<0.0001
Pillai's Trace	32, 516	0.7382	9.43	<0.0001
Population(Species)				
Wilks' Lambda	160, 2213.9	0.1135	4.02	<0.0001
Pillai's Trace	160, 2660	1.8292	3.72	<0.0001
BASED ON ORIGIN				
All data				
Species				
Wilks' Lambda	40, 486	0.1561	18.60	<0.0001
Pillai's Trace	40, 488	1.1872	17.82	<0.0001
Population(Species)				
Wilks' Lambda	420, 3596.8	0.0149	2.88	<0.0001
Pillai's Trace	420, 5240	3.3820	2.54	<0.0001
Subset 16 elements				
Species				
Wilks' Lambda	32, 494	0.2758	13.96	<0.0001
Pillai's Trace	32, 496	0.9412	13.78	<0.0001
Population(Species)				
Wilks' Lambda	336, 3205	0.0303	3.00	<0.0001
Pillai's Trace	336, 4192	2.8684	2.73	<0.0001

df, degrees of freedom
P, probability

Supplementary Table 8b. MANOVA to assess differences in seed mineral nutrition based on population from which seeds were collected. Wilks' Lambda and Pillai's Trace test statistics from MANOVAs performed within each species testing for population/origin effects on the seed elements. Separate analyses were performed for population and origin.

Source	df	Statistic value	F-Value	P-value
BASED ON POPULATION				
All data				
Ech Species				
Wilks' Lambda	40, 90	0.157	3.43	<0.0001
Pillai's Trace	40, 92	1.190	3.38	<0.0001
Ret Species				
Wilks' Lambda	160, 1276.4	0.020	5.52	<0.0001
Pillai's Trace	160, 1408	2.819	4.79	<0.0001
Subset 16 elements				
Ech Species				
Wilks' Lambda	32, 98	0.185	4.05	<0.0001
Pillai's Trace	32, 100	1.127	4.04	<0.0001
Ret Species				
Wilks' Lambda	128, 1260.2	0.045	5.30	<0.0001
Pillai's Trace	128, 1440	2.380	4.76	<0.0001
BASED ON Origin				
All data				
Ech Species				
Wilks' Lambda	100, 209.6	0.024	2.42	<0.0001
Pillai's Trace	100, 230	2.501	2.30	<0.0001
Ret Species				
Wilks' Lambda	320, 2079.7	0.003	3.89	<0.0001
Pillai's Trace	320, 2832	4.247	3.20	<0.0001
Subset 16 elements				
Ech Species				
Wilks' Lambda	80, 225.8	0.037	2.78	<0.0001
Pillai's Trace	80, 250	2.276	2.61	<0.0001
Ret Species				
Wilks' Lambda	256, 1925.1	0.008	4.00	<0.0001
Pillai's Trace	256, 2896	3.627	3.32	<0.0001

df, degrees of freedom
P, probability

Supplementary Table 9a. *Helicoverpa armigera* detached leaf assay.

Accession	Species	Remark	Damage Rating (1-9)		Larval Survival (%)		Larval Weight (g)		Host suitability index	
			30 DAE	60 DAE	30 DAE	60 DAE	30 DAE	60 DAE	30 DAE	60 DAE
Sirna_060	*C. reticulatum*		3.2	3.4	58	78	0.49	0.74	0.95	1.72
Karab_092	*C. echinospermum*		2.2	5.6	44	76	0.6	1.43	1.26	1.95
Ortan_066	*C. echinospermum*		5.6	6.2	72	82	1.06	1.43	1.37	1.91
Gunas_062	*C. echinospermum*		4.4	3.6	66	80	0.82	0.98	1.29	2.18
Egill_065	*C. reticulatum*		4.6	7.8	78	80	1.13	1.77	1.94	1.82
Kalka_064	*C. reticulatum*		4.6	6.8	82	86	1.32	1.26	2.38	1.59
Besev_079	*C. reticulatum*		3.8	3.4	76	68	1.49	0.85	2.97	1.7
Oyali_084	*C. reticulatum*		5.8	5.6	82	78	1.96	1.43	2.77	1.99
Deste_080	*C. echinospermum*		4.4	4.6	68	76	1.8	1.3	2.78	2.16
Derei_072	*C. reticulatum*		3.6	5	82	82	1.27	1.21	2.94	2.02
Derei_070	*C. reticulatum*		4.6	6.4	78	80	1.48	1.97	2.52	2.46
Bari2_072n2	*C. reticulatum*		4.6	4.6	68	82	1.7	1.38	2.53	2.47
CudiA_152	*C. reticulatum*		3	2.2	58	64	1.3	0.83	2.59	2.42
CudiB_022C	*C. reticulatum*		6	4.6	94	86	1.7	1.31	2.69	2.46
Kayat_077	*C. reticulatum*		3.8	5.2	64	90	2.14	1.05	3.67	1.84
S2Drd_065	*C. echinospermum*		5.2	6.8	70	88	2.39	1.9	3.26	2.45
Cermi_075	*C. echinospermum*		2.4	4.6	58	64	1.35	1.71	3.35	2.37
Bari1_092	*C. reticulatum*		6	4.6	75	84	2.46	1.51	3.08	2.75
Egill_073	*C. reticulatum*		4.4	5.4	76	94	1.65	1.67	2.91	2.93
Kesen_075	*C. reticulatum*		5.4	4.8	78	94	2.23	1.37	3.22	2.7
Sarik_067	*C. reticulatum*		6	5	96	96	1.93	1.47	3.12	2.83
**ICC506EB**	***C aerietinum***	**Resistant**	**2.6**	**3.2**	**70**	**80**	**1.21**	**1.1**	**3.33**	**2.76**
**IG 72953**	***C. reticulatum***	**Wild Resistant**	**3.4**	**5.6**	**68**	**94**	**1.8**	**1.5**	**3.63**	**2.5**
Besev_075	*C. reticulatum*		5.4	2.8	86	80	1.5	1.34	2.42	3.85
Bari3_072c	*C. reticulatum*		5.6	4	82	84	2.37	1.43	3.5	3.05
Bari3_106D	*C. reticulatum*		4.4	2.4	70	72	1.54	1.63	2.47	4.91
Bari3_100	*C. reticulatum*		4.2	4.8	76	74	2.57	1.94	4.75	3.02
**ICC3137**	***C. aerietinum***	**Susceptible**	**3.6**	**6.4**	**80**	**82**	**2.58**	**2.19**	**5.8**	**2.82**
Savur_063	*C. reticulatum*		3.8	5.2	68	90	2.75	2.2	4.96	3.8
**ICCV 95334**	***C. aerietinum***	**Susceptible**	**3**	**4.4**	**70**	**74**	**1.98**	**2.39**	**4.75**	**4.05**

DAE, days after exposure. Green, resistant checks; red, susceptible checks.

Supplemental Table 9b. *Helicoverpa armigera* nutritionally complete diet supplementation assay. Green, resistant checks; red, susceptible checks.

Accession	Species	Remark	Larval survival on 10th day (%)	Larval survival on 15th day (%)	10th day larval weight (mg)	15th day larval weight (mg)	Weight gain (mg)
Sirna_060	*C. reticulatum*		50	38	4	5.3	1.3
Karab_092	*C. echinospermum*		46	22	5.3	7	1.7
Ortan_066	*C. echinospermum*		62	54	10.8	25.6	14.8
Gunas_062	*C. echinospermum*		54	50	11.1	21.1	10.1
Egill_065	*C. reticulatum*		70	54	17.9	38.6	20.6
Kalka_064	*C. reticulatum*		74	66	9.4	19.2	9.9
Besev_079	*C. reticulatum*		84	78	18.2	83.7	65.5
Oyali_084	*C. reticulatum*		56	48	7.8	16	8.2
Deste_080	*C. echinospermum*		62	56	10.5	22.3	11.8
Derei_072	*C. reticulatum*		70	58	8.6	17.2	8.6
Derei_070	*C. reticulatum*		54	48	11	20.9	9.9
Bari2_072n 2	*C. reticulatum*		72	54	14.8	45.9	31.1
CudiA_152	*C. reticulatum*		44	40	9.2	14.9	5.7
CudiB_022 C	*C. reticulatum*		60	44	10.5	23.6	13
Kayat_077	*C. reticulatum*		64	58	17.1	30.8	13.7
S2Drd_065	*C. echinospermum*		48	46	8.2	13.4	5.2
Cermi_075	*C. echinospermum*		48	42	8.5	13.8	5.3
Bari1_092	*C. reticulatum*		84	78	18.4	85.6	67.2
Egill_073	*C. reticulatum*		54	48	6.5	9	2.5
Kesen_075	*C. reticulatum*		78	66	9.5	22	12.4
Sarik_067	*C. reticulatum*		74	68	10.3	20	9.7
**ICC506EB**	***C. aerietinum***	**Resistant**	**42**	**34**	**7.9**	**10.9**	**3**
**IG 72953**	***C. reticulatum***	**Wild Resistant**	**56**	**54**	**27.9**	**70.5**	**42.6**
Besev_075	*C. reticulatum*		64	56	7.7	13.2	5.5
Bari3_072c	*C. reticulatum*		42	42	9.7	21	11.2
Bari3_106D	*C. reticulatum*		52	50	10.1	20	9.9
Bari3_100	*C. reticulatum*		78	70	20.6	66.2	45.6
**ICC3137**	***C. aerietinum***	**Susceptible**	**94**	**90**	**23.1**	**75.1**	**52**
Savur_063	*C. reticulatum*		58	56	8.9	21.6	12.8
**ICCV 95334**	***C. aerietinum***	**Susceptible**	**86**	**78**	**23.9**	**74.6**	**50.7**

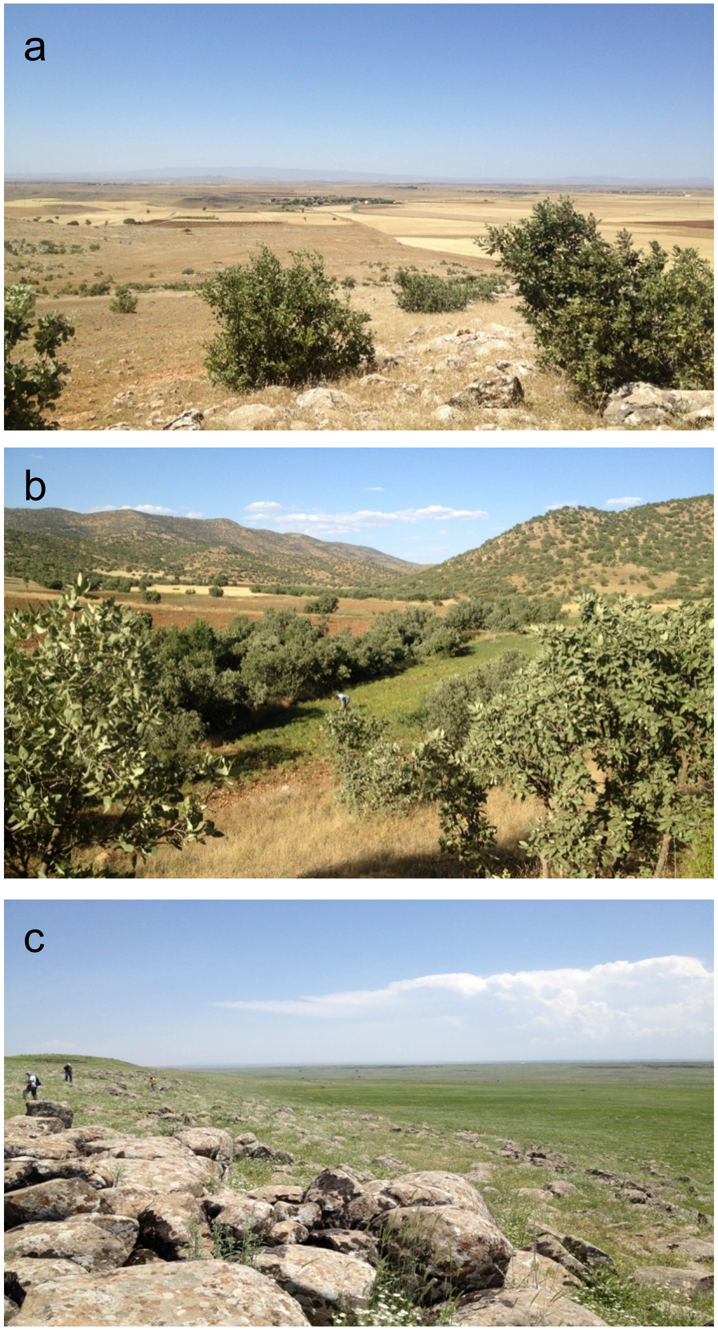

Supplementary Figure 1. Photo Montage of collecting and habitats. **a.** and **b.** depict *C. reticulatum* habitat, while **c.** is a *C. echinospermum* habitat. Images contributed by JB Eric von Wettberg.

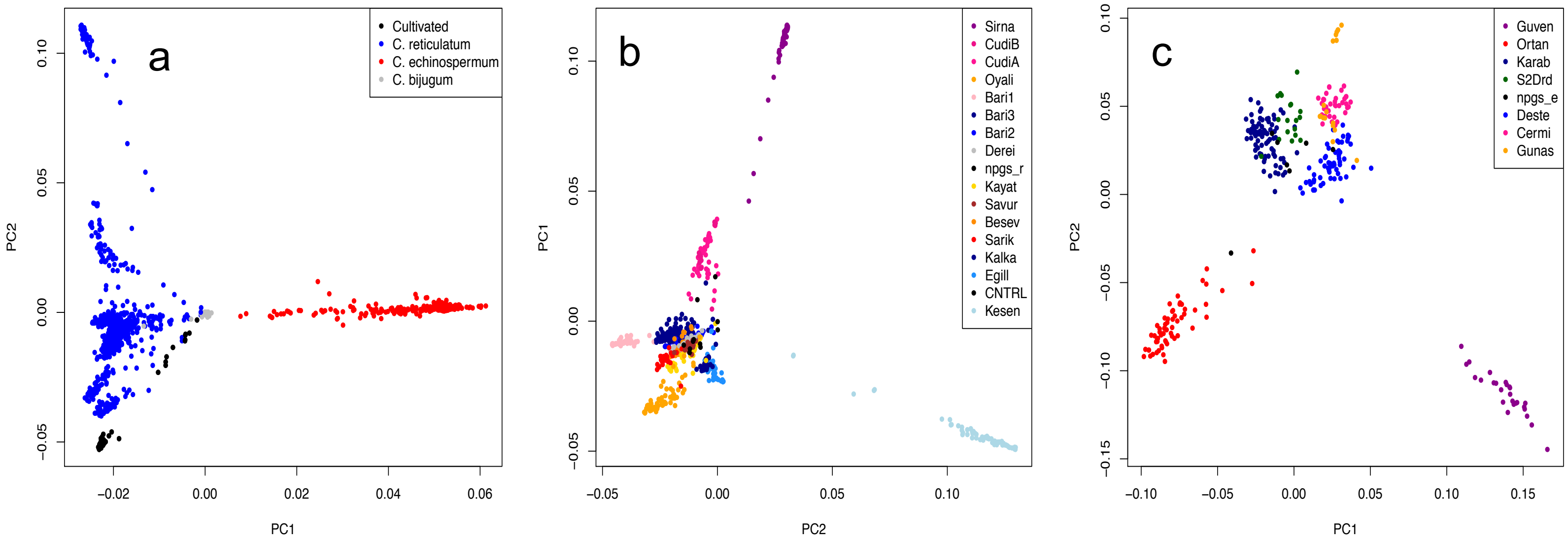

Supplementary Figure 2. Principal Coordinate analysis of 16,845 SNPs across 1,064 samples. **a.** Across samples of all three species. **b.** *Cicer reticulatum* samples alone. **c.** *Cicer echinospermum* samples alone.

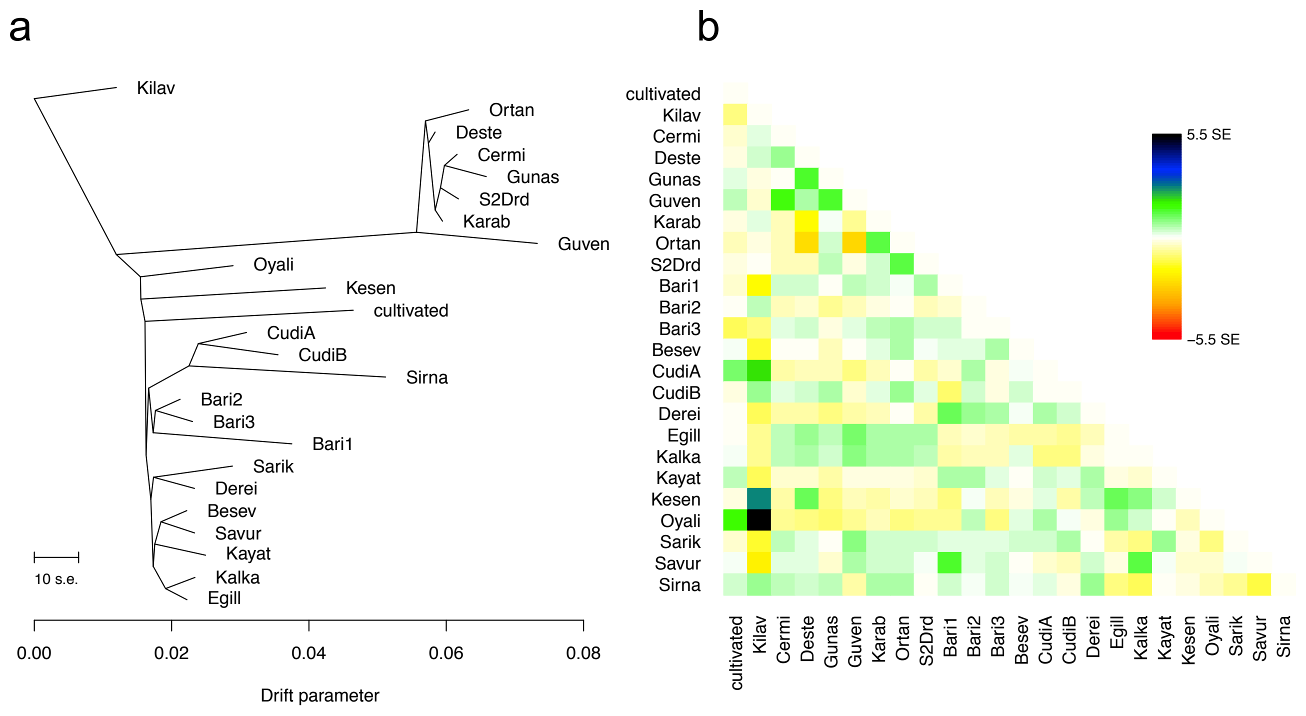

Supplementary Figure 3. Population relationships and admixture in *Cicer* species, part 1. **a.** Best fitted maximum likelihood tree displaying relationships between populations with no migration events. Migration edges are colored according to the proportion of source population ancestry found in the sink population. **b.** Plot of residuals for the tree depicted in **a**. The color palette depicts the residual covariance between each pair of populations divided by the average standard error across all pairs.

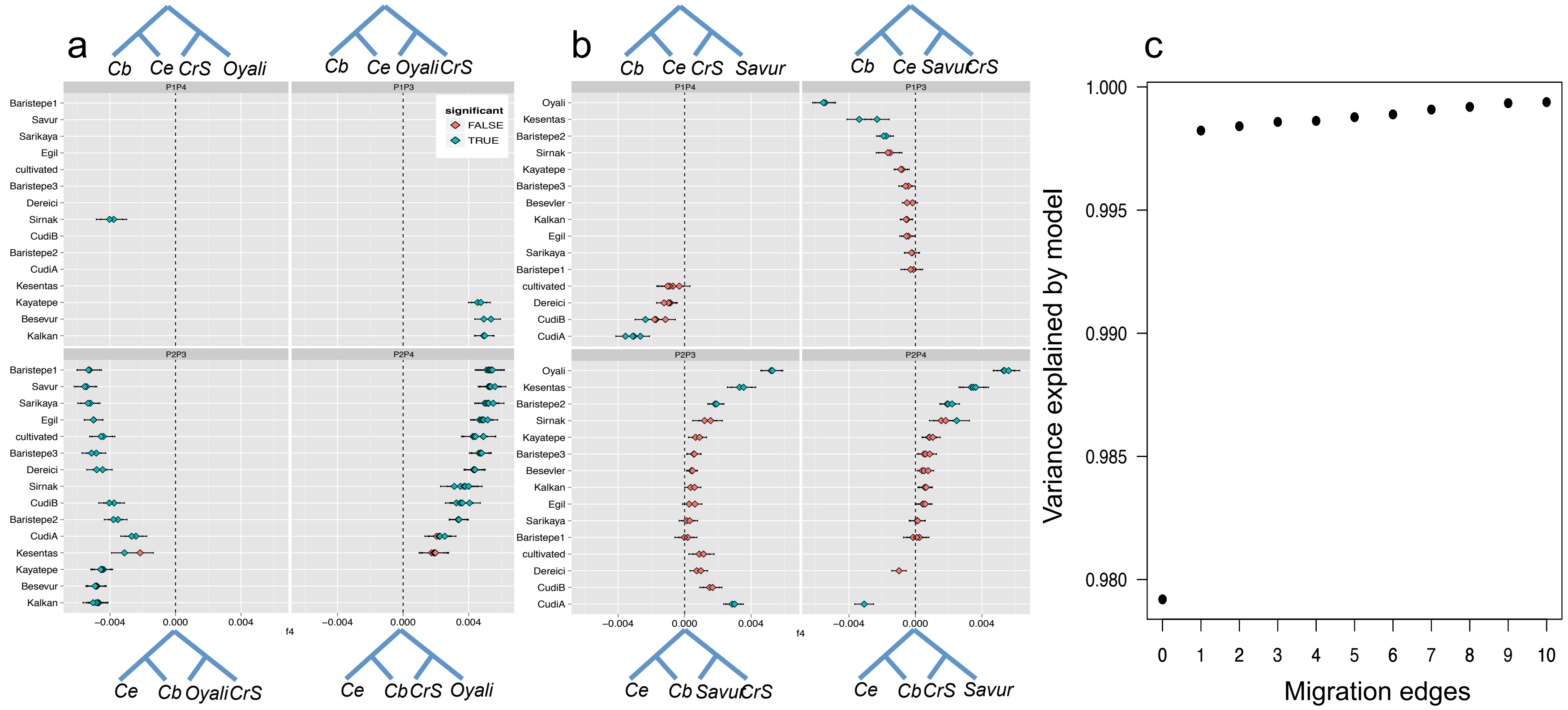

Supplementary Figure 4. Population relationships and admixture in *Cicer* species, part 2. The *f4* statistics from four-population tests, testing for gene-flow between **a.** Kilavuz (*C. bijugum*) and Oyali field sites, and between **b.** Kilavuz and Savur field sites. *Cb, C. bijugum*; *Ce, C. echinospermum*; *CrS, C. reticulatum*. **c.** Proportion of variance in sample covariance matrix that was accounted for by the fitted tree model covariance matrix, with models incorporating 0 through 10 migration events.

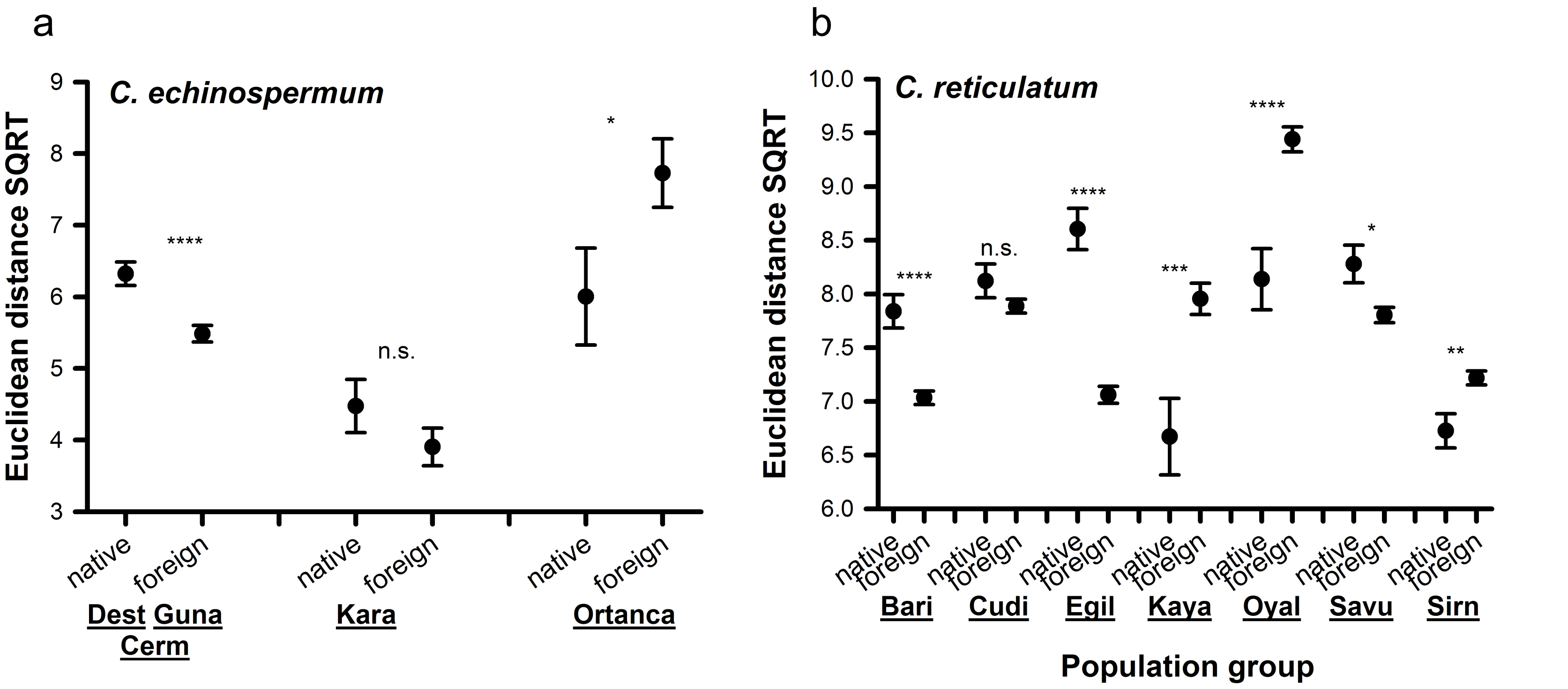

Supplementary Figure 5. Euclidean distance between color of seeds and color of soils among STRUCTURE defined populations and field sites, respectively. **a.** *Cicer echinospermum.* **b.** *C. reticulatum.* “Native” indicates the color distance within a field site, and “foreign” the color distance to other field sites.

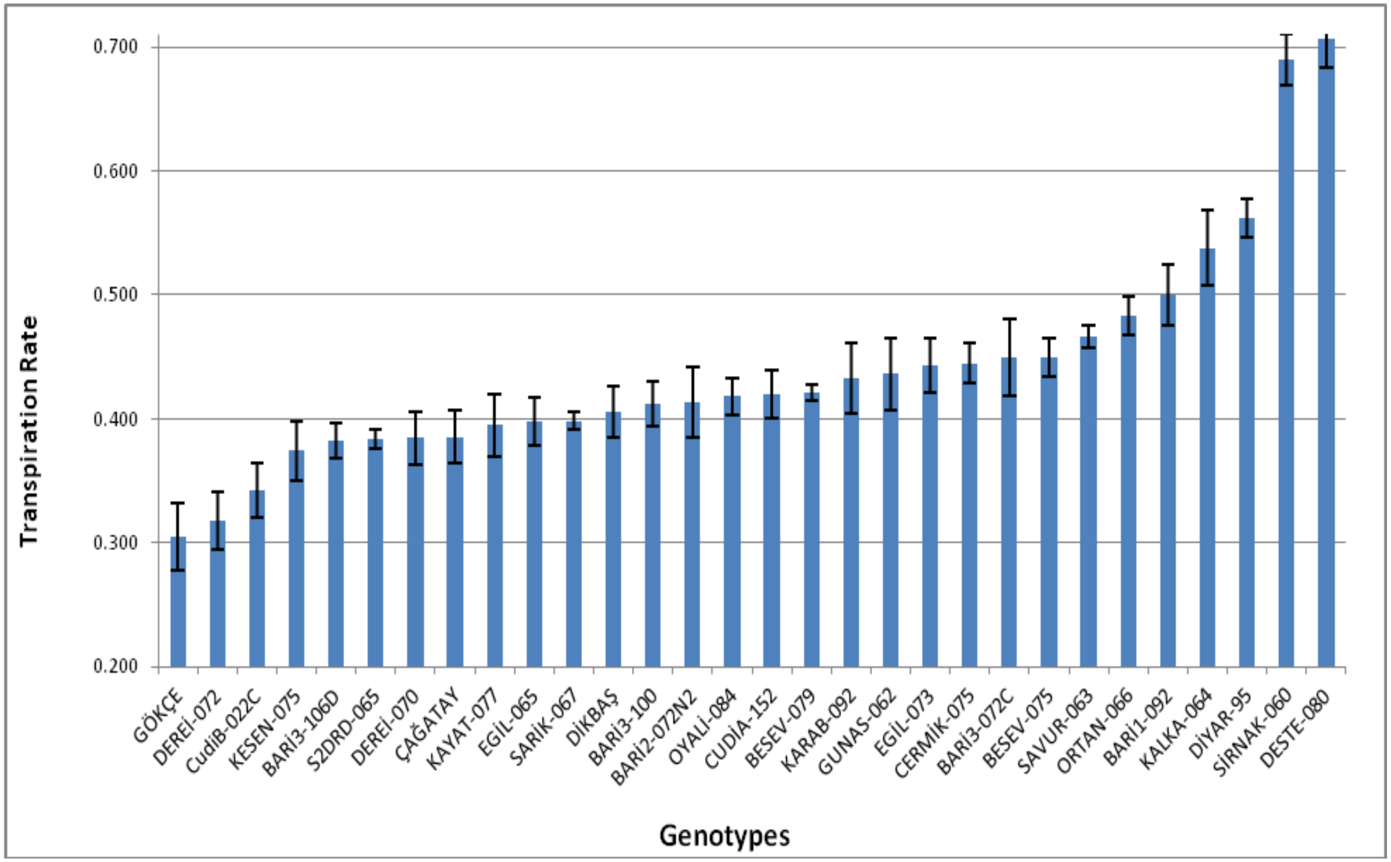

Supplementary Figure 6. Transpiration rate of 26 wild Cicer accessions and 4 cultivated chickpea varieties during a day-long VPD experiment. Error bars are standard deviation.

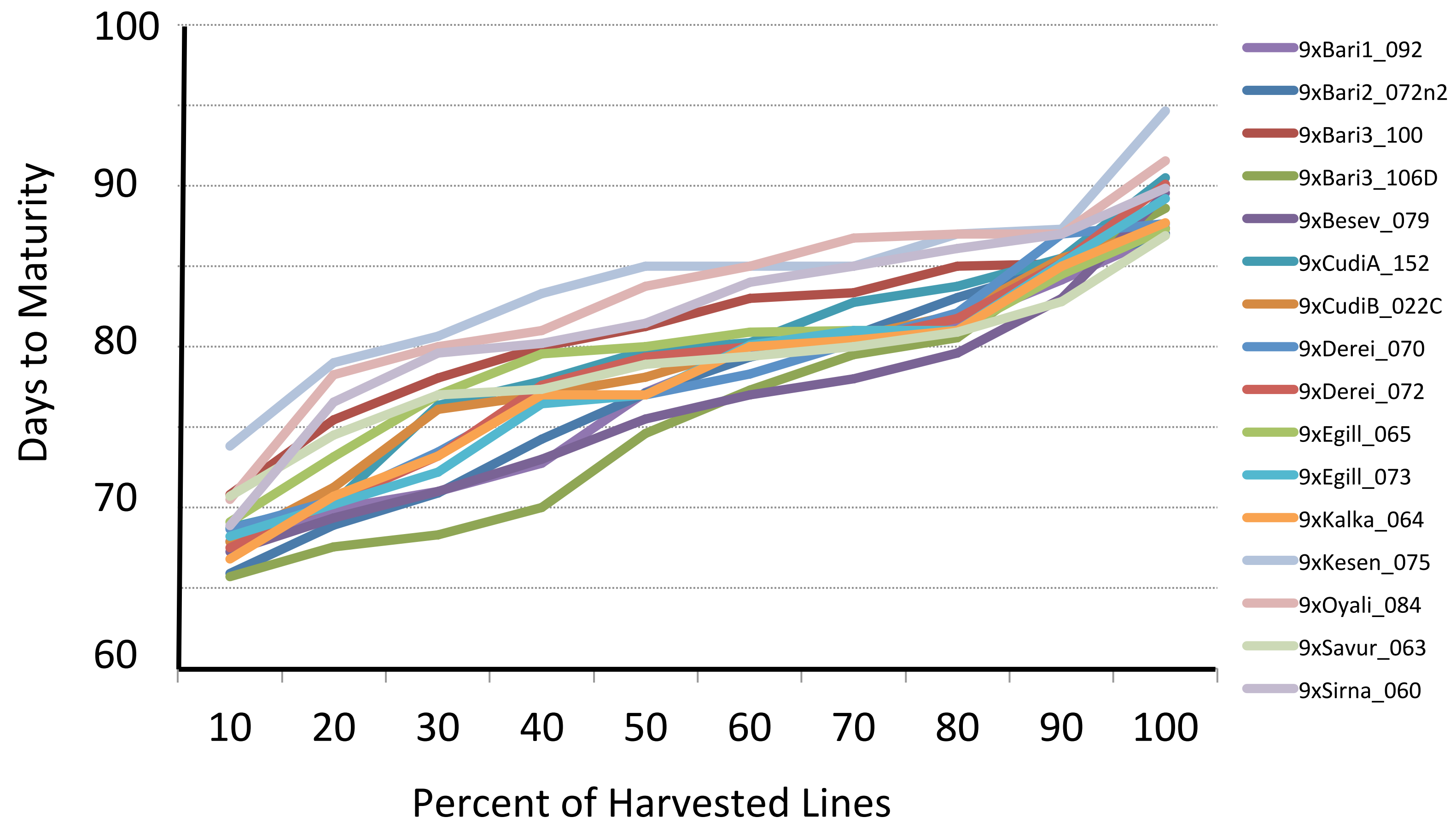

Supplemental Figure 7. Segregating maturity time variation in F2 populations. Shown are data for sixteen wild *C. reticulatum* accessions, each crossed into a common cultivated line (ICC 96029). Progeny segregate for an early flowering time locus from ICC 96029 which behaves in a partially dominant manner. Variation in the earliest maturing tail of the distribution reflects the background contribution of wild variation to early flowering imposed by the ICC 96029 allele.

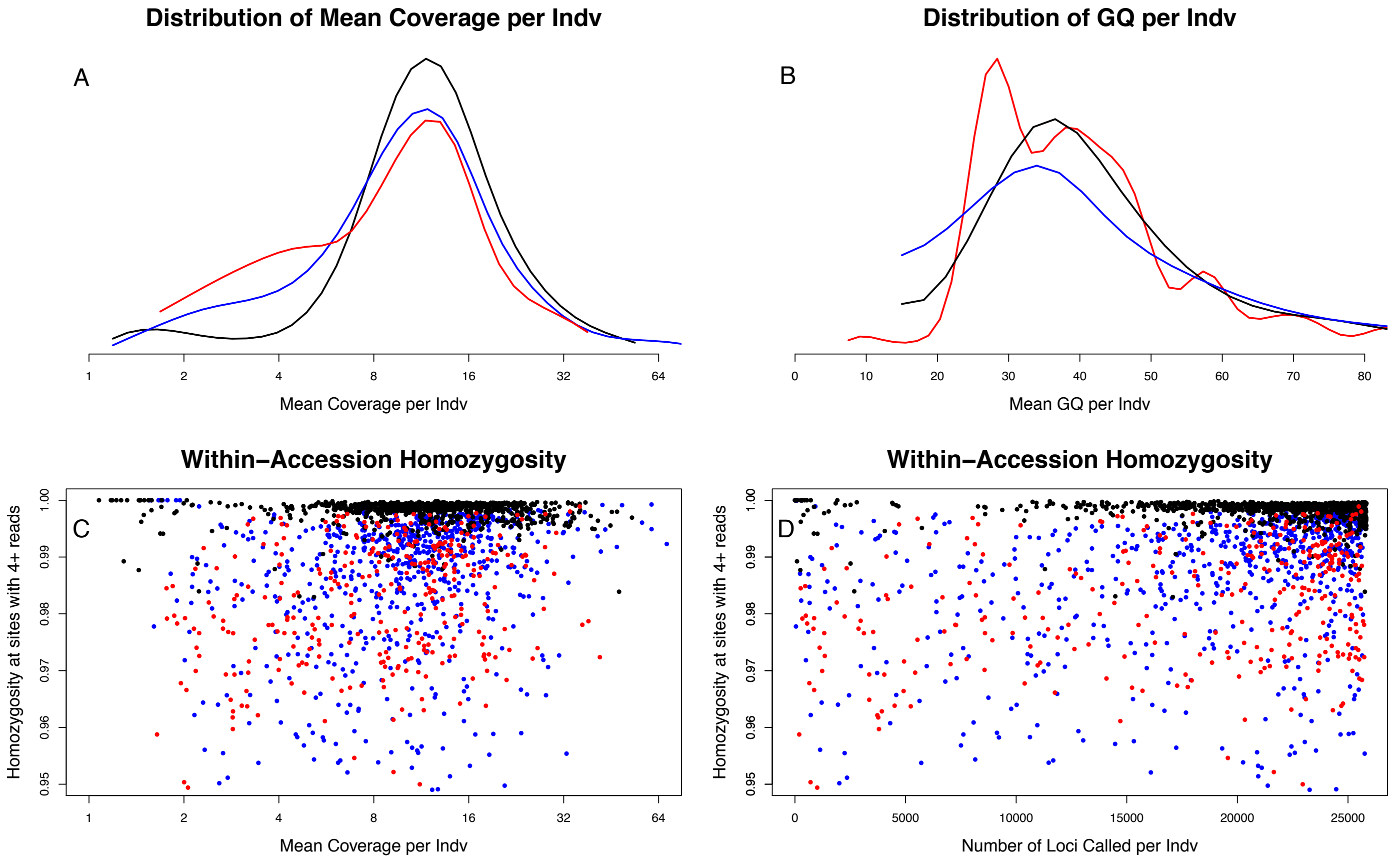

Supplementary Figure 8. Mapping summary of genome coverage, genotype quality, and observed homozygosity of GBS data across accessions. *C. arietinum* (black), *C. reticulatum* (blue), and *C. echinospermum* (red). Distributions of per-accession: **a.** mean coverage, and **b.** mean genotype quality across three *Cicer* species. Observed homozygosity calculated independently for all accessions at loci supported by at least four reads are not correlated with **c.** its mean coverage or **d.** the number loci called.
