# Supplementary material for: Ecology and genomics of an important crop wild relative as a prelude to agricultural innovation
Source: Nat Commun. 2018 Feb 13;9:649. doi: 10.1038/s41467-018-02867-z (PMC5811434; doi:10.1038/s41467-018-02867-z)
Supplement: Supplementary file 2 — Description of Additional Supplementary Files [file 41467_2018_2867_MOESM2_ESM.pdf]

Description of Additional Supplementary Files

File Name: Supplementary Data 1
Description: Plant accession metadata.

File Name: Supplementary Data 2
Description: Seed micronutrient analysis.
